# Genes involved in auxin biosynthesis, transport and signalling underlie the extreme adventitious root phenotype of the tomato *aer* mutant

**DOI:** 10.1007/s00122-024-04570-8

**Published:** 2024-03-08

**Authors:** Zoltan Kevei, Eduardo Larriba, María Dolores Romero-Bosquet, Miriam Nicolás-Albujer, Tomasz J. Kurowski, Fady Mohareb, Daniel Rickett, José Manuel Pérez-Pérez, Andrew J. Thompson

**Affiliations:** 1 Centre for Soil, AgriFood and Biosciences, Cranfield University, College Road, Bedfordshire, MK43 0AL UK; 2 Instituto de Bioingeniería, Universidad Miguel Hernández, 03202 Elche, Spain; 3 Syngenta Crop Protection, Jealott’s Hill International Research Centre, Bracknell Berkshire, RG42 6EY UK

## Abstract

The use of tomato rootstocks has helped to alleviate the soaring abiotic stresses provoked by the adverse effects of climate change. Lateral and adventitious roots can improve topsoil exploration and nutrient uptake, shoot biomass and resulting overall yield. It is essential to understand the genetic basis of root structure development and how lateral and adventitious roots are produced. Existing mutant lines with specific root phenotypes are an excellent resource to analyse and comprehend the molecular basis of root developmental traits. The tomato *aerial roots* (*aer*) mutant exhibits an extreme adventitious rooting phenotype on the primary stem. It is known that this phenotype is associated with restricted polar auxin transport from the juvenile to the more mature stem, but prior to this study, the genetic loci responsible for the *aer* phenotype were unknown. We used genomic approaches to define the polygenic nature of the *aer* phenotype and provide evidence that increased expression of specific auxin biosynthesis, transport and signalling genes in different loci causes the initiation of adventitious root primordia in tomato stems. Our results allow the selection of different levels of adventitious rooting using molecular markers, potentially contributing to rootstock breeding strategies in grafted vegetable crops, especially in tomato. In crops vegetatively propagated as cuttings, such as fruit trees and cane fruits, orthologous genes may be useful for the selection of cultivars more amenable to propagation.

**Supplementary Information:**

The online version contains supplementary material available at 10.1007/s00122-024-04570-8.

## Introduction

Most dicot plants have root systems that consist of at least two different types of roots; the primary root (PR), which is established during embryogenesis, and the lateral roots (LRs), which develop de novo from root pericycle initials (Du and Scheres 2018). LRs enhance horizontal soil exploration and greatly contribute to the ability to outcompete neighbouring plants when exploiting the same soil niche (Doussan et al. 2009). Plants can also form adventitious roots (ARs) from non-root organs, such as the hypocotyl, stems or leaves, either naturally or in response to different environmental stresses or physical damage, such as wounds (Bellini et al. 2014). The root system architecture (RSA) is defined by the number, shape and spatial arrangement of the PR, LRs and ARs which, in turn, determine not only the volume of the soil to be explored, but also the ability of the plants to use efficiently the available water and nutrients. Breeding for favourable RSA traits has progressed slowly so far, as root phenotyping is challenging, expensive and time-consuming, especially in field trials (van der Bom et al. 2020).

Some crops can only be maintained in a genetically uniform state by vegetative propagated due to their breeding systems, and often vegetative propagation relies on formation of ARs from cuttings; however, the genetic capacity for AR formation varies and breeders must select lines that can readily be propagated by cuttings. For example, elite apple cultivars are typically propagated clonally using ARs produced from stem cuttings (Díaz-Sala 2021), but some cultivars propagate very poorly through formation of ARs from cuttings and can only be propagated effectively through grafting (Webster 1995). In another example from Rosaceae, raspberry rooted stolons produce new shoots (“suckers”) from which cuttings are rooted for propagation (Qiu et al. 2017); cultivars with a high capacity to form ARs are easier to propagate and are preferred by breeders.

Special types of ARs, known as crown roots and shoot-borne roots, can arise from the basal region of the stem and represent the main root system in monocot plants, such as maize and rice (Marcon et al. 2013). In rice, *DEEPER ROOTING1* (*DRO1*) emerged as a key regulatory gene that modulates the growth angle of LRs and ARs and directly contributes to increased root system depth, thus having a positive impact on rice yield in drought conditions (Uga et al. 2013). *DRO1* orthologs have been identified in a wide range of other plant species, including both monocots and dicots, and in all cases the conservation of DRO1 function has been confirmed (Guseman et al. 2017). Moreover, a higher number of crown roots in rice cultivars resulted in a shallower root depth, which enhanced phosphorous uptake from low-phosphate soils (Sun et al. 2018). Therefore, crops with large number of ARs increase topsoil exploration in different plant species and thus can improve nutrient acquisition, growth, and yield where nutrients are limiting.

In the last two decades, most fresh market tomatoes have been produced as grafted plants where rootstocks provide resistance to soil-borne diseases and confer the desirable level of vigour and vegetative-reproductive balance to the scion; increasingly they are also the subject of investigations to mitigate against abiotic stresses (Schwarz et al. 2010). This has led to extensive research to understand how to generate the best scion and rootstock combinations to increase quality and yield under stress (Albacete et al. 2015). For marker-assisted rootstock breeding (Thompson et al. 2017), there is an obvious need to investigate the genetic regulation of tomato RSA, accelerated by the comprehensive and accessible datasets of genome sequences from different tomato species, cultivars and landraces (Fernandez-Pozo et al. 2015). A collection of predominantly monogenic tomato inbred mutants is also available to perform forward genetic studies (C.M. Rick Tomato Genetics Resource Centre, TGRC). There is a group of mutant lines with characteristic root phenotypes (Kevei et al. 2022) which serve as a valuable source to analyse and understand the molecular basis of root developmental traits.

Although roots arising from the hypocotyl are commonly referred to as ARs, in the case of tomato, genetic evidence has demonstrated that roots that arise from the basal region (defined as the lower 1 cm of the hypocotyl and the upper 1 cm of the primary root) are distinct from both ARs and LRs. Therefore, they have been named “basal roots” (Zobel et al. 1975). The basal roots were found to contribute the largest proportion of the total root biomass in field trials, representing between 61 and 85% of the total root dry mass across 23 cultivars (Stoffella 1983). This highlights the developmental and agronomic significance of basal roots and their relationship to ARs.

Tomato can produce ARs from undifferentiated callus or from reprogrammed cells, mostly in hypocotyl, leaf and stem tissues (Verstraeten et al. 2014), where overlapping, but different sets of genes and cells are induced for the development of LRs or ARs (Shaar-Moshe and Brady 2022). A TGRC root mutant line, *aerial roots* (*aer*), has numerous ARs on the main stem (Philouze 1971), and plentiful, early formation of AR primordia under flooding stress, resulting in ethylene insensitivity and better adaptation to flooding (Vidoz et al. 2016). Compared to control genotypes, polar auxin transport (PAT) has been shown to be blocked from younger to more developed parts of the stem in *aer*, resulting in numerous ARs in the basal stem region (Mignolli et al. 2017).

The plant growth hormone auxin has been widely studied for its multiple effects on plant and fruit development (Godoy et al. 2021; Gomes and Scortecci 2021), it also plays a fundamental role in the initiation and development of ARs in various species (Bellini et al. 2014; Gonin et al. 2019). During the early steps of AR formation, the accumulation of indole-3-acetic acid (IAA) occurs in specific cell types by PAT and generates local auxin biosynthesis, conjugation and degradation gradients (Lakehal and Bellini 2019). Indole-3-butyric acid (IBA) is also used for clonal propagation as the conversion of IBA to IAA promotes AR production from stem cuttings (Frick and Strader 2018). Studies have also revealed the crucial role of auxin in initiating AR development in tomato stem cuttings where auxin carriers and symporters were mainly induced during the initiation and extension stages of AR development (Guan et al. 2019). It was also shown that the expression of distal auxin transport upon hypocotyl wounding of the tomato cultivar Micro-Tom (MT) is required for AR induction at the basal cut site, where cell cycle reactivation of neighbouring cells initiates the development of ARs (Alaguero-Cordovilla et al. 2021). More recently, molecular cloning of the classical *rosette* (*ro*) mutation in tomato, possessing severely reduced internodes and complete sterility, revealed the role of the tomato gene orthologous to *BIG*/*TRANSPORT INHIBITOR RESPONSE 3* (*TIR3*) in the activity of the PIN-FORMED (PIN) auxin transport proteins: the *ro* phenotype resulted in reduced auxin transport rates and absence of ARs in stems (Modrego et al. 2023).

Here, we present a comprehensive genetic analysis of the *aer* mutant showing genomic variation at several auxin-related loci on different chromosomes, confirming the expected polygenic nature of the *aer* phenotype (Mignolli et al. 2017). Our study provides evidence that increased expression of local auxin synthesis and auxin regulated genes induce the initiation of AR primordia in tomato stems of *aer*. This is a trait that could be used to support breeding strategies of tomato and other crops to improve nutrient uptake and yield and also to promote the efficient propagation of outbreeding species.

## Materials and methods

### Plant material and growth

Tomato (*Solanum lycopersicum* L.) cultivar Ailsa Craig (AC) and Ailsa Craig carrying an introgression from *Solanum peruvianum* on chromosome 9 with the resistance allele of the *Tobacco mosaic virus resistance-2*^*a*^ locus (AC-*Tm-2*^*a*^) were used as parental lines for F_2_ crosses (Figures S1, S2). The *aerial root* mutant seeds (*aer*, accession number LA3205) were provided by the Tomato Genetics Resource Center (TGRC, University of California, Davis). Seed accession numbers with prefix “WSS” were generated and archived at Cranfield University.

Tomato seeds were germinated, and plants were grown for AR phenotyping and seed bulking as described (Kevei et al. 2022). Phenotype was scored in fully developed plants by counting AR numbers on the stem from the soil level to a height of 35 cm.

For the root penetration assay, the plants were grown in approximately 2 kg of Sinclair All Purpose Growing Medium Compost (LBS Worldwide Ltd, Lancashire, UK) inside assembled pipes separated by a 140-micron aperture made of 0.065 mm stainless steel metal mesh, as shown in Figure S3. The mesh was purchased from Plastok Meshes and Filtration (Birkenhead, UK). To maintain high moisture levels for the penetrating roots, the plastic saucers, which are similar in size to the pipes, were filled with water absorbent AquaMat Capillary Matting (LBS). The appropriate drainage was ensured by perforating the saucers and the capillary matting.

### Plant growth conditions for wound induced AR phenotype

Seeds of AC, AC-*Tm-2*^*a*^ and *aer* lines were surface-sterilized in 5% (v/v) commercial bleach for 10 min and rinsed thoroughly with sterile distilled water (four times). Seeds were then transferred to 120 × 120 mm square Petri plates containing 75 mL of sterile germination medium composed of half-strength Murashige and Skoog basal salt (MS) medium (Duchefa Biochemie, The Netherlands), 2.5 g L^−1^ Gelrite (Duchefa Biochemie), 0.5 g L^−1^ 2-(N-morpholino) ethane sulfonic acid (Duchefa Biochemie) and 2 mL L^−1^ 1 × Gamborg B5 vitamin solution (Duchefa Biochemie), at pH 5.8. The plates were incubated overnight in darkness at 4 °C, and afterwards, placed in a growth cabinet in a 16 h light period (average photosynthetic photon flux density of 50 µmol m^−2^ s^−1^) at 26 ± 1 °C, and 8 h darkness at 23 ± 1 °C.

Germinated seedlings with primary roots > 4 mm 5 days after sowing (DAS) were transferred to new plates in a nearly vertical orientation. At 7 DAS, once young tomato seedlings were at the 100–101 growth stages (fully expanded cotyledons and first leaf ~ 0.5 cm; (Feller et al. 1995)), the formation of ARs was induced by removing with a sharp scalpel the whole root system 2–3 mm above the hypocotyl-root junction (0 days after whole root excision; 0 DAE). To minimize the effect of inner auxins, 2/3 parts of the cotyledons were cut. The shoot explants were transferred to 65 × 150 mm (diameter × height) glass jars with 50 mL of sterile regeneration medium composed of half-strength MS medium (Duchefa Biochemie, The Netherlands), 20 g L^−1^ sucrose (Duchefa Biochemie), 2.5 g L^−1^ Gelrite (Duchefa Biochemie), 0.5 g L^−1^ 2-(N-morpholino) ethane sulfonic acid (Duchefa Biochemie) and 2 mL L^−1^ 1 × Gamborg B5 vitamin solution (Duchefa Biochemie), at pH 5.8. The medium was supplemented with Yucasin DF and L-Kynurenine in dimethyl sulfoxide at 50 µM each. Two jars were assayed per genotype and treatment.

### DNA extraction, KASP and InDel genotyping

Genomic DNA was extracted from young cotyledons/leaves, and the KASP/KBD assays were performed as described (Silva Ferreira et al. 2018). KBD assays were developed by LGC (Teddington, UK) based on the provided SNP and flanking sequence data (Table S1). The KASP genotyping results were analysed in CFX96 qPCR machines (CFX Connect) using the “Allelic Discrimination” feature of CFX manager software (BioRad, Watford, UK). InDel markers were generated by PCR amplification and agarose gel electrophoresis of shorter DNA fragments (under 500 bp) containing the allelic size differences. The selected primers pairs and InDel positions are shown in Table S2.

### NGS genomic data generation and sequence analysis

Genomic DNA from AC, AC-*Tm-2*^*a*^ and *aer* were extracted using the DNeasy plant mini kit (Qiagen; Manchester, UK), according to the manufacturer’s instructions. They were sequenced using Illumina HiSeq X platforms (paired end 2 × 150 bp; PE150). The data comprised 419,548,170 (AC), 389,240,368 (AC-*Tm-2*^*a*^) and 400,917,844 (*aer*) 100 bp reads representing ~ 42×, ~ 39 × and  ~ 40 × average read depths, respectively. Data are available from SRA accession of PRJNA882342 (NCBI). Reads were aligned to the SL2.50 (Heinz 1706) reference genome and variants were called using the “Alpheus” pipeline (Miller et al. 2008). AC possessed 173,793, AC-*Tm-2*^*a*^ had 1,139,329 and *aer* carried 634,456 sequence variants compared to Heinz 1706. The resulting VCF files were analysed by the Integrative Genomics Viewer (IGV) (Robinson et al. 2011), and KASP markers were designed for the genetic mapping of *aer* based on the detected polymorphisms.

### Bulk segregant analysis

Bulk segregant analysis (BSA) was performed to investigate genetic regions potentially associated with the *aer* phenotype. Whole genome sequencing low quality reads from AC, AC-*Tm-2*^*a*^ and *aer* were removed using Trimmomatic (Bolger et al. 2014). Pre-processed reads were aligned to the reference tomato genome (*S. lycopersicum*, version SL2.50) using the Burrows–Wheeler Aligner (BWA; version 0.7.7) software settings (Sirén et al. 2014). Duplicates were marked using Picard tools (MarkDuplicates function), then variants were identified (for each sample) using Genome Analysis Toolkit (GATK4, version 4.1.9.0; HaplotypeCaller function in GVCF mode) (McKenna et al. 2010). Pooled pre-processed reads from the bulks were aligned to the reference genome using BWA-MEM followed by variant calling using the GATK pipeline. BSA was performed by following the SNP-index method as representation of the frequency of the of alternate (alt) allele in a specific locus of the bulked sample population (Schneeberger 2014). SNP-index values at each position were calculated by dividing the number of reads supporting the alt allele by the total number of reads corresponding to that locus. The total is obtained for the reference (ref) allele and the alt allele. SNP-index were then plotted using a sliding window across all chromosomes. SNP-index values are expected to be randomly distributed around 0.5 (i.e., 50% of reads supporting each position are expected from both parents) for most parts of the genome where the loci are not linked to the causal mutation, while linked loci and the actual mutation region are expected to have a SNP-index value closer to 1.

### RNA-seq and bioinformatics analyses

For expression analyses, root (total root) and stem (up to 30 mm above soil) tissues of four weeks old plants (when AR primordia were already visible) were harvested, and the total RNA was extracted by Spectrum Plant Total RNA Kit (Sigma). The RNA sequencing (Illumina Sequencing, PE150) was performed at Novogene-Europe (Cambridge). Raw reads were purified and aligned to *Solanum lycopersicum* genome SL4.0 (Heinz 1706) assembly using HISAT2 (Sirén et al. 2014). Gene counts were obtained using StringTie (Pertea et al. 2015). Raw counts of ITAG4 genes were normalized to CPM (counts per million), removing genes with less than 0.5 CPM in three samples, and differentially expressed genes (DEGs) with fold change >|2| (FDR < 0.05) were obtained using voom/limma package (Law et al. 2014) implemented in DEGUST (Powell 2019). Tomato orthologs of *Arabidopsis thaliana* genes were obtained as described (Larriba et al. 2021). Heatmaps and hierarchical clustering were performed using Morpheus online tool of Broad Institute.

Flanking regions from ORF start point of *SBRL* and *SlTAR2b* from *aer*, SL2.50 and SL4.0 genomes were retrieved using BEDTools suite (Quinlan and Hall 2010), and from *S. lycopersicum* SL2.50 and SL4.0 genome assemblies using Blast in SolGenomics (Fernandez-Pozo et al. 2015). DNA alignments were performed using *Clustal Omega* (Madeira et al. 2022). Putative transcription factor (TF) binding sites in the *SBRL* and *SlTAR2* promoters were identified using the *Binding Site Prediction* tool in PlantRegMap using a 120 bp genomic DNA sequence flanking the identified SNPs (Tian et al. 2020).

### Genotype-by-sequencing (GBS) and QTL analyses

GBS was deployed to uncover polymorphism distribution in the F_2_ population derived from the AC × *aer* cross (Figure S2**)**. The GBS was performed by LGC (Teddington, UK) with the following parameters: The library was generated with MslI digestion (insert size: ~ 220 bp) and NextSeq 500 PE250 kit was used to produce the sequence data on NovaSeq sequencer, which resulted in total number of 20,387 SNPs across all samples. The QTL analysis of the AR phenotype was performed using TASSEL software (Bradbury et al. 2007) using the mixed linear model with 1000 permutations and a *P* value of 0.005.

## Results

### The* aer* phenotype

**Fig. 1:**
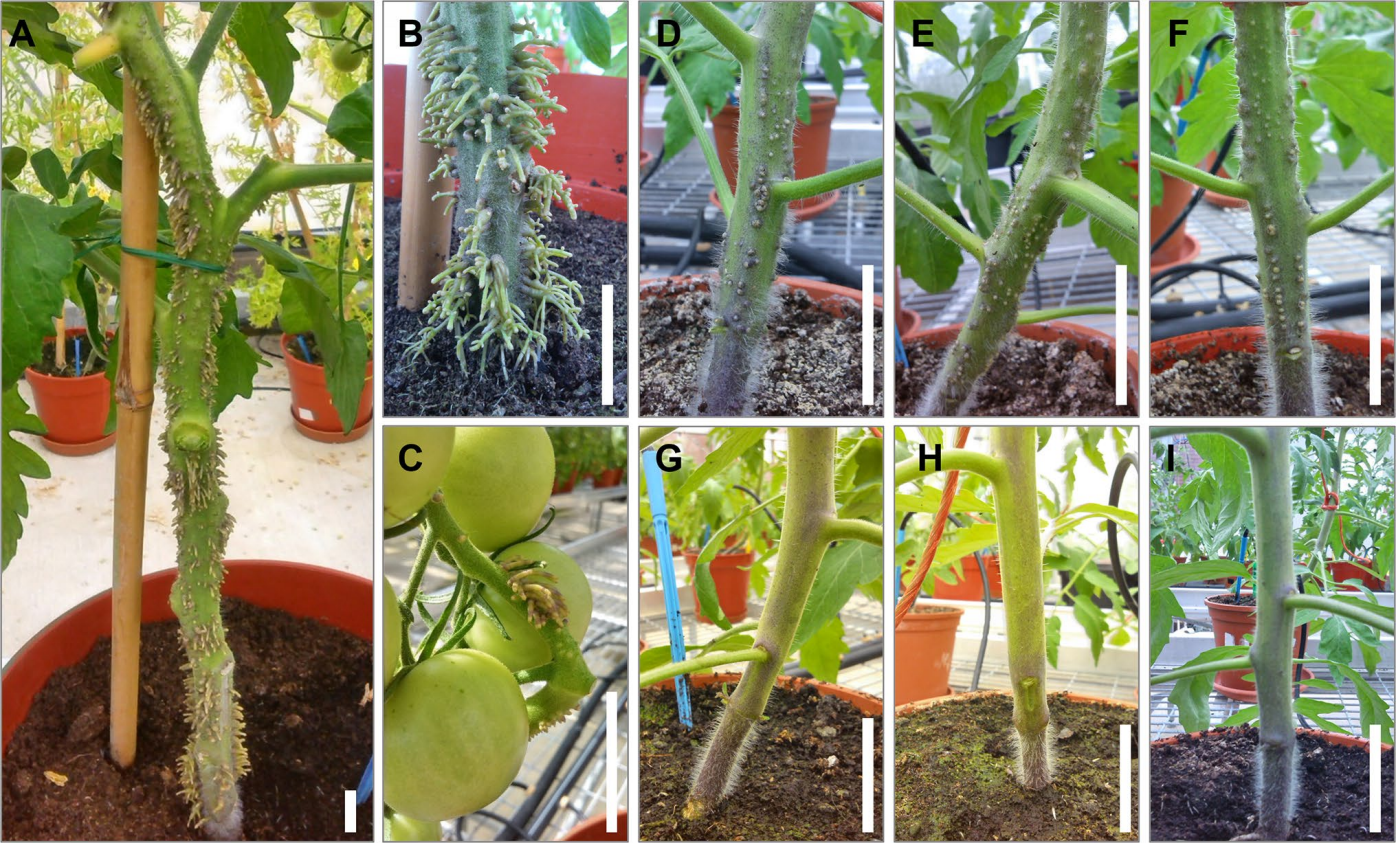
*aer* and AC phenotypes. Adventitious roots (ARs) appear along the entire stem on fully developed *aer* plants (**A**), but more prominently at the basal part of the stem (**B**) and may also occur at the trusses (**C**). For the bulked segregant analyses (BSA), plant lines of the F_2_ population with apparent AR^+^ (**D**, **E**, **F**) and AR^−^ (**G**, **H**, **I**) phenotypes were selected. Scale bars: 30 mm

The principal feature of *aer* tomato plants is the presence of plentiful AR primordia on the surface of epicotyls and hypocotyls of 4 week-old seedlings (Mignolli et al. 2017) and the increased AR vigour upon flood stress (Vidoz et al. 2016). While AC plants showed no ARs in standard glasshouse growth conditions, the fully developed *aer* lines produced ARs along the whole stem (Fig. 1A) with a more noticeable phenotype at the basal part (Fig. 1B). We also observed extensive ARs on the first fruit trusses (Fig. 1C).

**Fig. 2:**
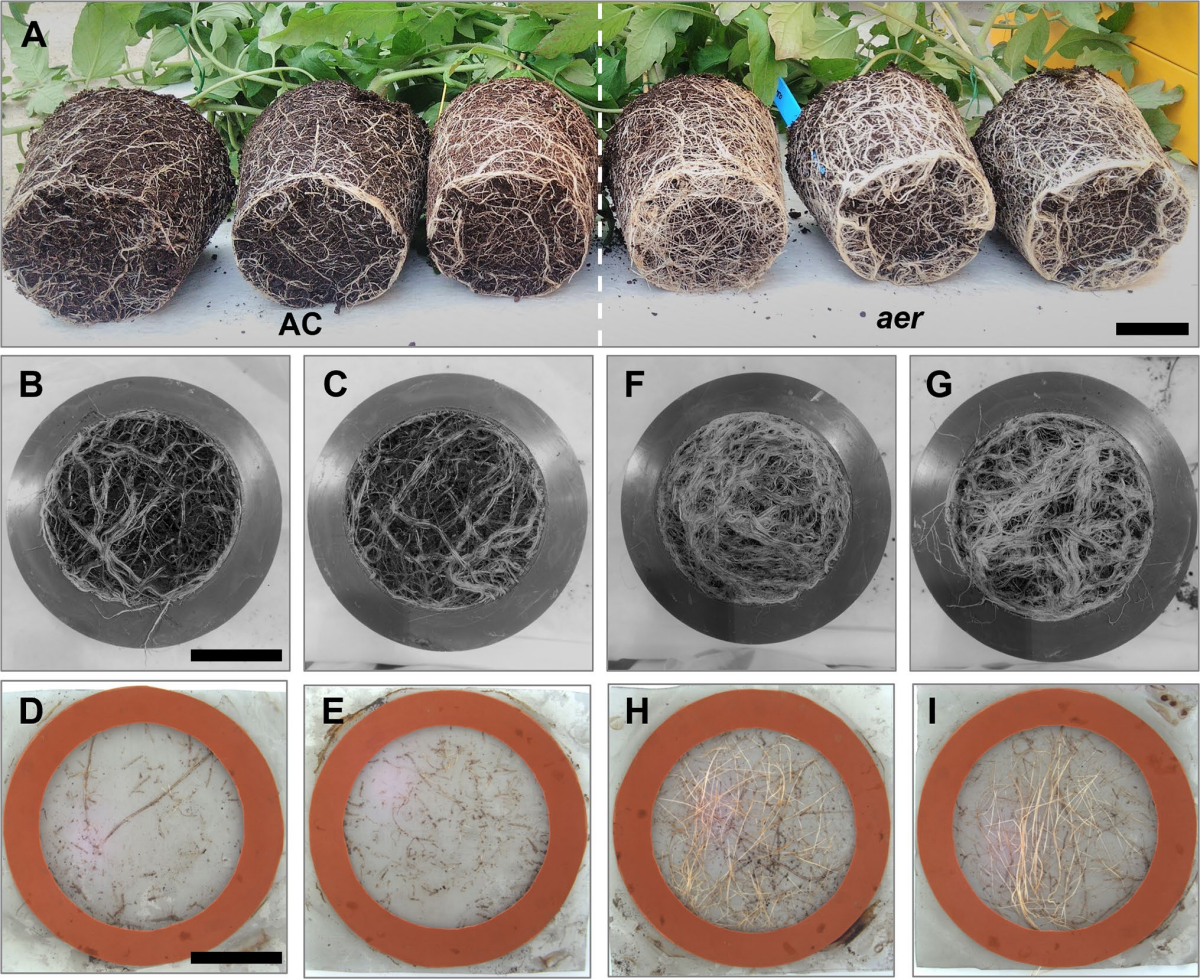
Root systems of AC and *aer* lines. Triplicates of 6-week-old AC and *aer* lines showed differences in root mass growing in pots (**A**). The root penetration assay (Fig. S3) showed a large variation in root mass between genotypes, with AC having fewer roots formed above (**B**,** C**) and below (**D**, **E**) the separation mesh compared to that of *aer* above (**F**, **G**) and below (**H**, **I**) the mesh. Scale bars: 50 mm

Since LR growth is closely related to AR development (Bellini et al. 2014), we also examined the root system of the *aer* lines. For phenotype comparison, we used the AC cultivar which produces significantly less ARs than *aer* (Vidoz et al. 2016). The 8-week-old AC and *aer* lines showed variation in root mass, *aer* had more LRs in the pots than AC by visual inspection (Fig. 2A). To confirm quantitatively the presence of increased root mass, we developed a “root-penetration” assay where roots need to grow through a separating metal mesh for root counting (Figure S3). On both sides of the dividing mesh the root mass of the two genotypes was strikingly different. AC has less roots formed above (Fig. 2B, C) and under (Fig. 2D, E) the separating mesh compared to that of *aer* above (Fig. 2F, G) and under the split region (Fig. 2H, I). The difference of penetrating root numbers was confirmed by root counting, and *aer* has significantly more pass-through roots than AC (Figure S4).

### Wound-induced AR phenotype

**Fig. 3:**
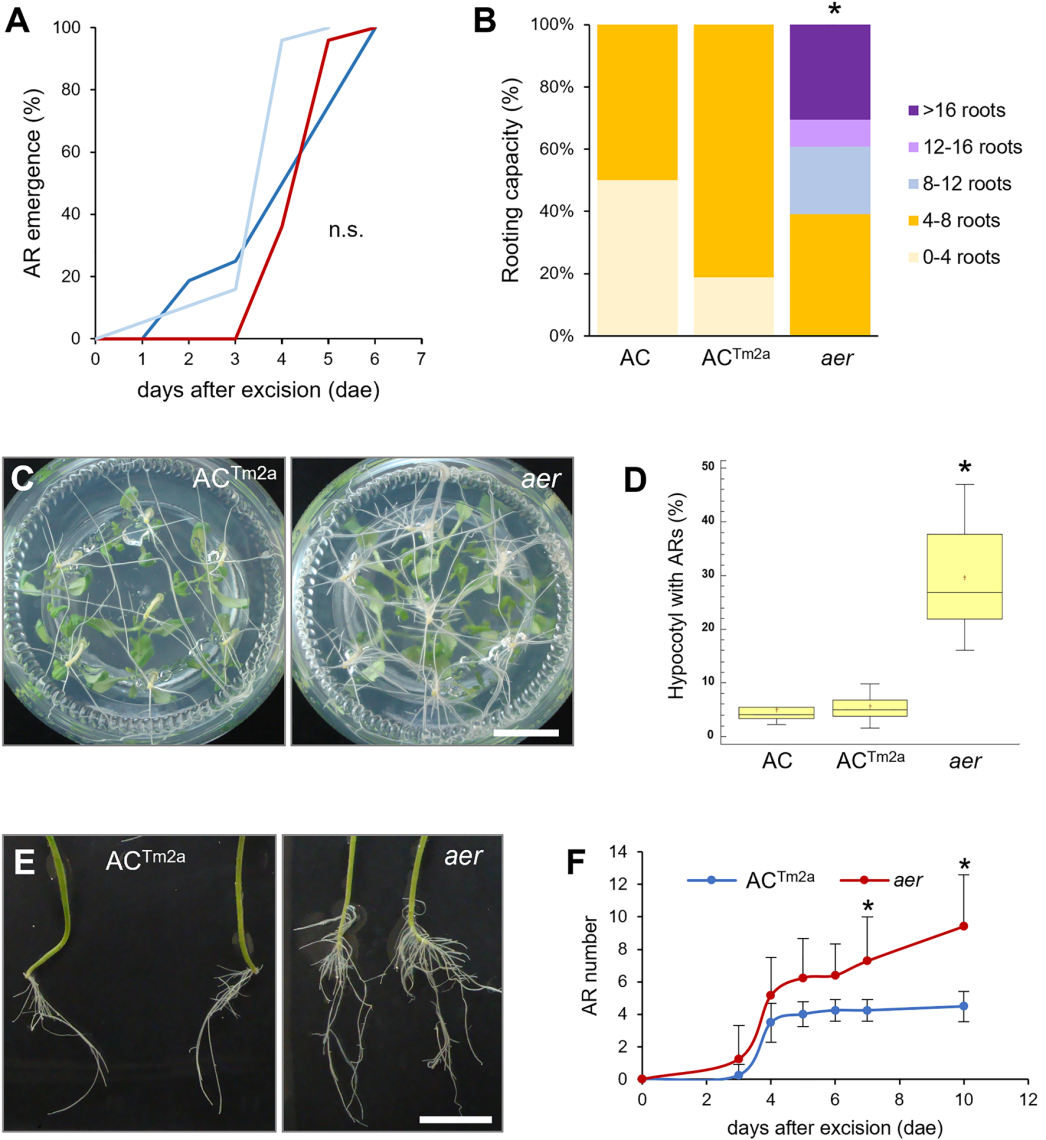
Wound-induced AR formation in *aer* shoot explants. (**A**) AR emergence of AC (light blue), AC-*Tm-2*.^*a*^ (dark blue) and *aer* (red) explants. (**B**) Rooting capacity of shoot explants at 10 days after whole root excision of young tomato hypocotyls (DAE). (**C**) Images of growth of shoot explants in glass jars for 10 days. (**D**) Percentage of hypocotyl length with ARs at 10 DAE. (**E**) Representative images of rooted shoot explants at 10 DAE. (**F**) Increase in the number of ARs in shoot explants over the studied time. Asterisks in **B**, **D** and **F** indicate significant differences (*p* value < 0.01) between genotypes; n.s., non-significant differences. Scale bars: 25 mm (**C**, **E**)

Wounding is a key trigger to produce ARs from shoot explants (Steffens and Rasmussen 2016), therefore we tested the *aer* capacity for AR production in young tomato hypocotyls after whole root excision (Alaguero-Cordovilla et al. 2021). We found no differences in the timing for AR emergence between *aer*, AC and AC-*Tm-2*^*a*^ (Fig. 3A) which lines were used for the genetic crosses. However, the *aer* line produced a significantly higher number (*p* value < 0.01) of ARs than in the AC and AC-*Tm-2*^*a*^ lines (Fig. 3B, C). We noticed that the AR formative region in the basal region of the hypocotyl near the wounding was larger in the *aer* line than in the AC and AC-*Tm-2*^*a*^ lines (Fig. 3D, E). Besides, the *aer* line produced ARs in the basal region of the hypocotyl during an extended period, which was not the case for the AC and AC-*Tm-2*^*a*^ lines (Figure 3F).

### Genetic mapping of* aer*

To analyse the genetic background of the extreme AR phenotype, we crossed *aer* with AC-*Tm-2*^*a*^ to create an F_2_ mapping population (Figure S1). Bulked segregant analyses (BSA) of the F_2_ population (Michelmore et al. 1991) was performed to identify the causative locus/loci of *aer*. For the BSA, we bulked two extreme phenotypes of 6 week-old plants; one bulk possessed apparent AR phenotype (AR^+^) lines (Fig. 1D–F), however they had less ARs than the original *aer* parent. The opposite bulk possessed no ARs (AR^−^) of any kind (Fig. 1G–I). From a total of 200 evaluated F_2_ lines, the genomic DNA of 45 AR^+^ and 52 AR^−^ lines were separately pooled for the genetic analyses (Figure S1). Whole genome resequencing (SL2.50) was performed on both parental lines and both pools to execute the BSA.

The BSA results of the AR^+^ phenotype revealed a major contributing genome region on chromosome 9 (Figure S5). The SNP zygosity index of the contrasting pools further delimited the potential causative region between 4.0 and 68.0 Mbp, where the average zygosity index of the AR^+^ pool was higher than 0.75 (Figure S6). A second locus was detected on chromosome 4, although the BSA signal showed moderated division compared to that of chromosome 9 locus. This second large causative region was located between 6.7 and 64.0 Mbp of chromosome 4 (Figure S7).

### Fine mapping of chromosome 9 locus

**Fig. 4:**
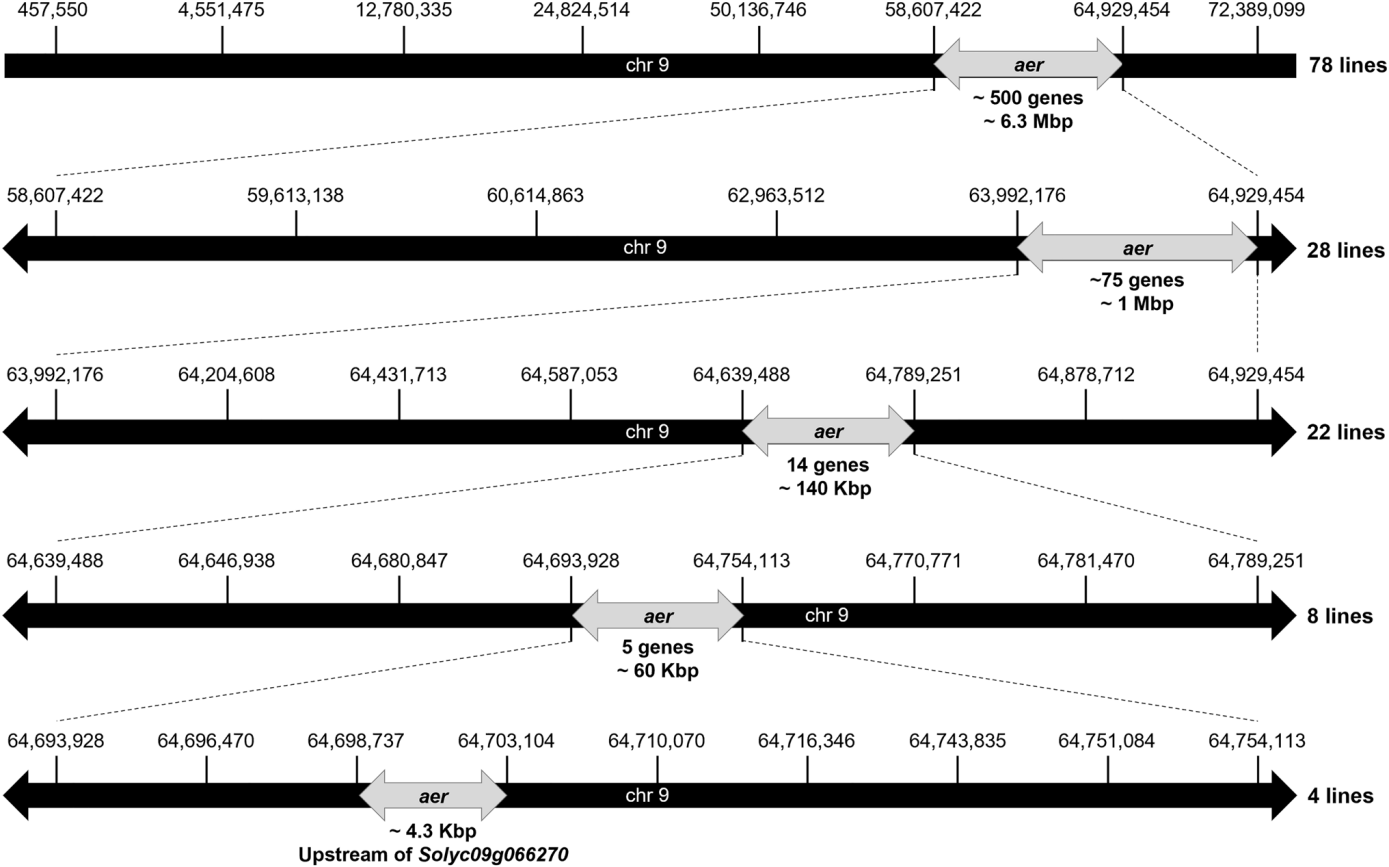
Schematic summary of the recombinant mapping process of the *aer* locus on chromosome 9. The SL2.50 positions (bp) of SNPs/InDels used for KASP and InDel marker design are indicated. The number of recombinant lines delimiting the actual causative regions during the subsequent mapping steps are marked. The size of the reduced mapping regions and their gene numbers are indicated. Further details of the mapping process are described in the text

We used the whole genome resequencing data of the parental *aer* and AC-*Tm-2*^*a*^ to design KASP DNA markers (Table S1) for the recombinant mapping of chromosome 9 *aer* locus. We started the mapping with an extended *aer* region on chromosome 9, two flanking markers were designed at 457, 550 and 72,389,099 bp (SL2.50) to isolate recombinant lines along chromosome 9 (Fig. 4). Seventy-eight recombinants were isolated after screening 350 F_2_ lines with these markers. They were further tested with additional inner markers and phenotyped for the presence of ARs. The phenotype data and the allelic segregation of these markers (4,551,475; 24,824,514; 50,136,746; 58,607,422; 64,929,454 bp) delimited the causative region to 6.2 Mbp containing approximately 500 genes (Fig. 4). Additional four inner KASP markers were generated (59,613,138; 60,614,863; 62,963,512; 63,992,176 bp) to further reduce the causative region in 28 recombinants of the initial 78 lines. This resulted in a ~ 1 Mbp causative region possessing 75 genes (Fig. 4). The isolated recombinants showed no further recombination across this region, therefore 600 additional F_2_ lines from the same mapping population were screened with the 63,992,176 and 64,929,454 bp markers to find recombinants in this region. The resulting 22 new recombinant lines were phenotyped for ARs and additional markers were designed to genotype them in three successive stages along the ~ 1 Mbp region. Firstly, six new InDel markers on the 64,204,608; 64,431,713; 64,587,053; 64,639,488; 64,789,251; 64,878,712 bp (Table S2) were generated and tested, which shortened the causative region to ~ 140 Kbp including 14 genes (Fig. 4). Next, 6 more InDel markers were designed (64,646,938; 64,680,847; 64,693,928; 64,754,113; 64,770,771; 64,781,470 bp) and tested on the eight lines possessing recombination across the 140 Kbp region (Table S3). The allele and phenotype segregation led to further reduction of the causative region to 60 Kbp possessing only five genes inside (Fig. 4). Lastly, with the help of seven additional InDel and KASP markers (64,696,470; 64,698,737; 64,703,104; 64,710,070; 64,716,346; 64,743,835; 64,751,084 bp), the four remaining recombinant lines within the 60 Kbp region revealed a ~ 4.3 Kbp causative region between 64,698,737 and 64,703,104 bp. This was the upstream region of the *Solyc09g066270* gene (Fig. 4), which codes for LOB DOMAIN-CONTAINING PROTEIN 29 (LBD29) (Fernandez-Pozo et al. 2015) and has been recently named SHOOTBORNE ROOTLESS (SBRL) (Omary et al. 2022).

### Expression pattern of* SBRL*

**Fig. 5:**
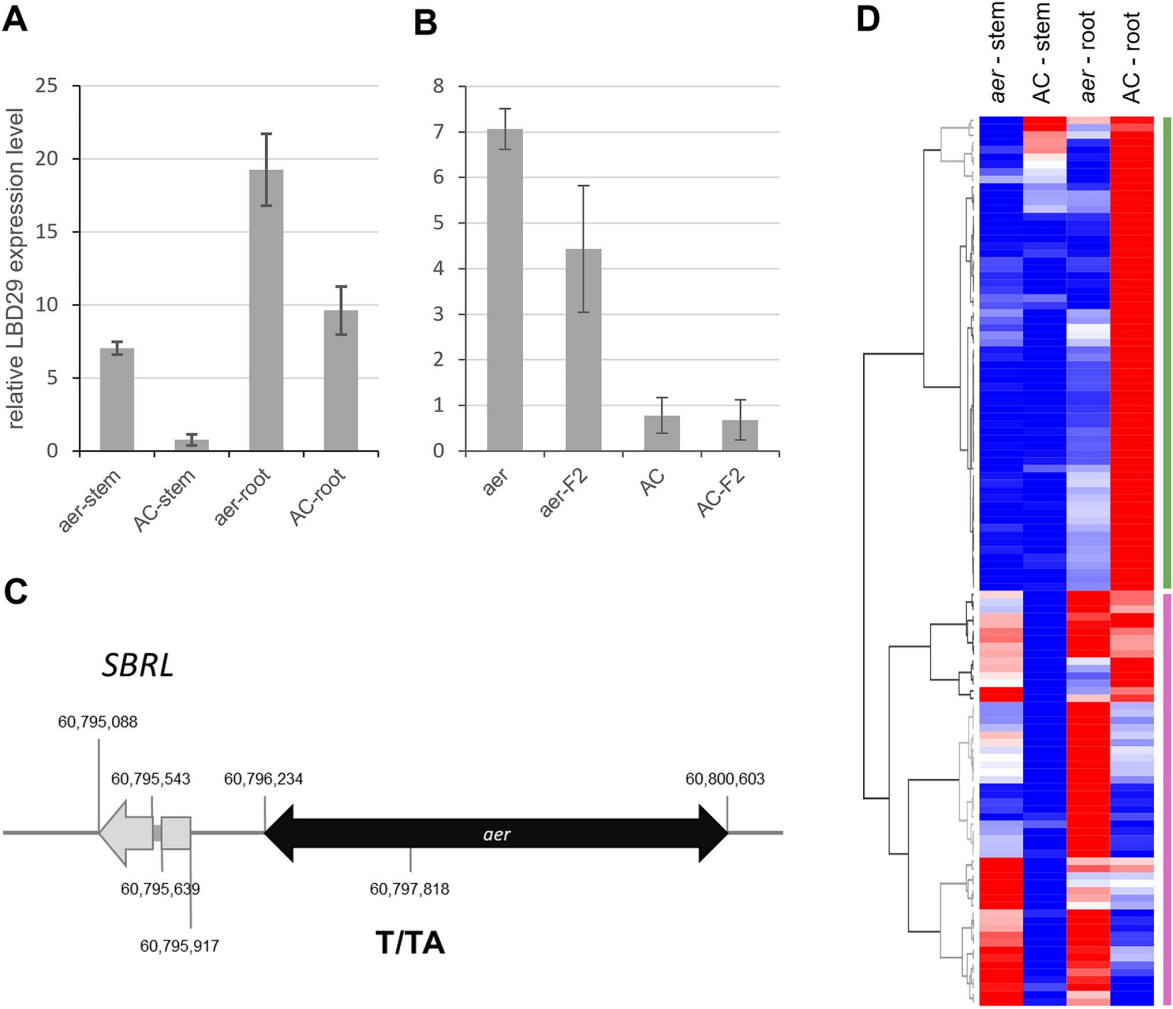
*SBRL* as a candidate gene for the AR^+^ phenotype in *aer*. **A**–**B** Expression of *SBRL* in stem and roots. The expression was measured in three biological samples for each genotype. They were normalized to the expression level of *SlACTIN2* (*Solyc03g078400*) as a constitutive control (*p* value < 0.01). Standard error bars are indicated. **C** Promoter region of *SBRL* including the candidate polymorphisms for the QTLs in this region (SL4.0). **D** Hierarchical clustering of the average expression of 54 DEGs in both stem and root tissues of *aer* lines with respect to AC

We investigated the expression pattern of the *SBRL/LBD29* gene in the two parental genotypes and in selected F_2_ lines with AR^+^ and AR^−^ phenotypes to pursue the potential impact of the mapped promoter region. Initially, we compared the RNA-seq data from root and stem tissues of *aer* and AC-*Tm-2*^*a*^, *aer* showed significantly increased *SBRL* expression both in stem and root tissues, it had approximately 10- and twofold average increase in stem and roots, respectively (Fig. 5A). *aer* and AC-*Tm-2*^*a*^ has unlinked genetic backgrounds to perform appropriate gene expression comparison, therefore randomly selected F_2_ lines, which were fixed for parental genotypes in the causative region, were tested for the *SBRL* expression. The result showed that the *aer* allele of the recombinant line indeed promotes increased *SBRL* transcript level (~ sevenfold) in the basal stem region (Fig. 5B).

The start of the causative upstream region of *SBRL* is separated by 317 bp from the actual start codon of the gene (Fig. 5C). It contained no unique sequence variation compared to the 150 Tomato Resequencing Project (Aflitos et al. 2014) and Tomato 360 Resequencing Project (Lin et al. 2014), however, 1964 bp upstream from the start codon an “A” insertion (SL4.0_chr09: 60,797,824 bp) was found in *aer*, which only occurs in the *S. galapagense* and *S. pimpinellifolium* lines among the re-sequenced wild tomato species (Aflitos et al. 2014). We investigated whether this upstream sequence variation of the *SBRL* gene can affect its transcriptional regulation, the in silico analyses uncovered a potential TF binding site in this region (see Materials and Methods). This insertion in *aer* introduced a putative binding site for *Solyc11g008560*, encoding the AP2-like ethylene-responsive transcription factor PLETHORA2 (Aida et al. 2004), as well as for *Solyc06g062520*, encoding a C2C2-DOF transcription factor (Yanagisawa 2004) with a yet unidentified function in *A. thaliana*.

To investigate the SBRL-dependent expression changes that occur in the *aer* phenotype, we searched for the tomato putative orthologues of the LBD29 targets based on available Arabidopsis RNA-seq data (Xu et al. 2018). We found 341 putative orthologs of LDB29 targets which showed measurable expression in *aer* and AC tissues, of which 197 were found deregulated in stems and/or roots of the *aer* lines (71 DEGs in stem, 72 in roots and 54 shared; Table S4). Hierarchical clustering of 54 deregulated genes in *aer* stem and roots as regards those in AC were further studied (Fig. 5D and Table S4). One of the significantly upregulated targets in *aer* tissues was *Solyc11g013310*, which encodes a putative LAX3 auxin influx carrier that promotes emergence of both LRs and ARs in *A. thaliana* (Porco et al. 2016; Lee et al. 2019).

### Chromosome 4 loci for* aer*

**Fig. 6:**
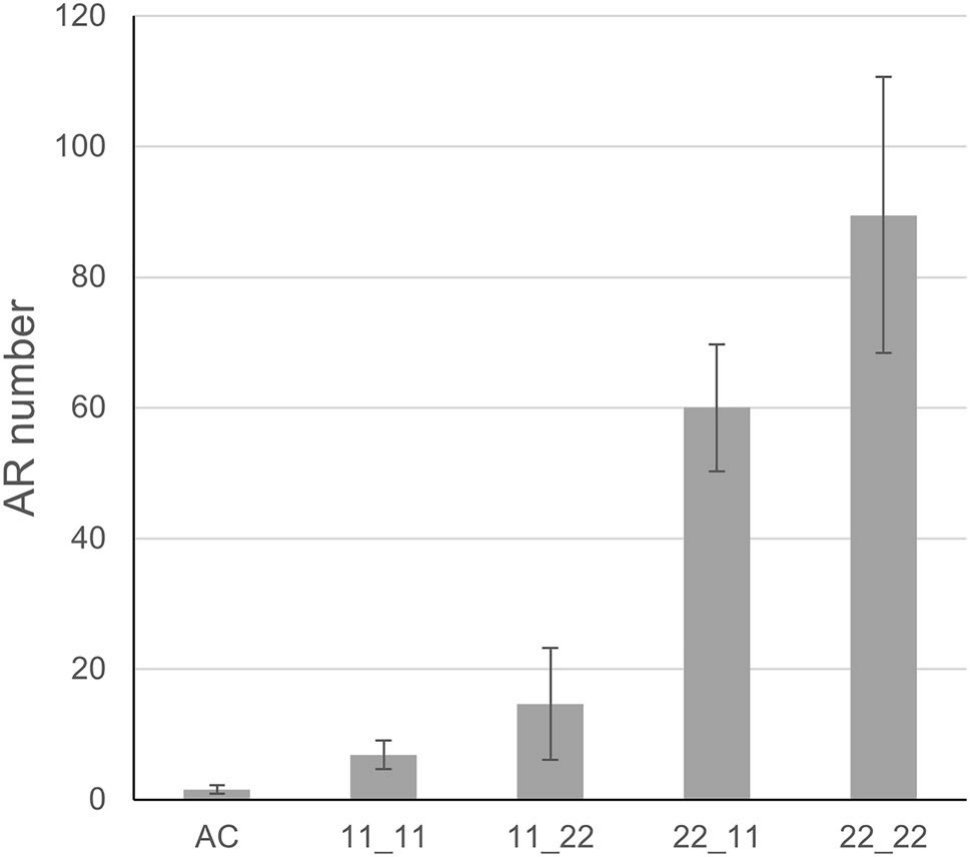
Combined effect of chromosome 4 and 9 alleles for the *aer* phenotype. Genotype 11 (homozygous allele for AC) and 22 (homozygous allele for *aer*) are indicated on chr4 and chr9 with the order of the “chr4_chr9” alleles on the graph. Bars represent average values with standard error (*p* value < 0.01)

The previous studies on *aer* (Mignolli et al. 2017) and the decreased number of ARs of the F_2_ recombinants from the *aer* × AC-*Tm-2*^*a*^ cross suggested that more than one gene is involved in the extreme AR phenotype. The BSA indicated an additional locus on chromosome 4 which might increase the number of ARs (Figure S7). To analyse the promoting effect of chromosome 4 loci on the *aer* phenotype, F_2_ lines of an AC × *aer* cross (Figure S2**)** possessing homozygous AC or *aer* alleles in the investigated chromosome 4 and 9 regions were selected by KASP markers and were phenotyped for AR numbers (Table S5). For the large chromosome 4 region, two flanking markers developed from the BSA result (Table S1), chr04-6.7 (at 6,783,452 bp in SL2.50) and chr04-64.0 (at 64,028,212 bp) were jointly used to select the homozygous alleles for the causative region. For chromosome 9, the *SBRL* linked chr09-*SBRL* marker (at 64,716,346 bp) was used for line selection (Table S1). The results showed that the *aer* allele on chromosome 4 is indeed positively contributing to the increased AR phenotype (Fig. 6). Among the four allele combinations, the highest AR numbers were detected when both causative chromosome 4 and 9 regions carried the *aer* allele. However, this allele combination has still produced significantly lower AR numbers than the original *aer* line, which possessed ~ 6 times more ARs on the similar stem part (Table S5). Beside this observation, the increased AR numbers in the lines with homozygous AC alleles both on chromosomes 4 and 9 and the large variation of AR numbers in the different allele groups (Table S5) clearly indicated a further locus or loci which influence the large AR number of *aer*.

### Third* aer* loci on chromosome 3

**Fig. 7:**
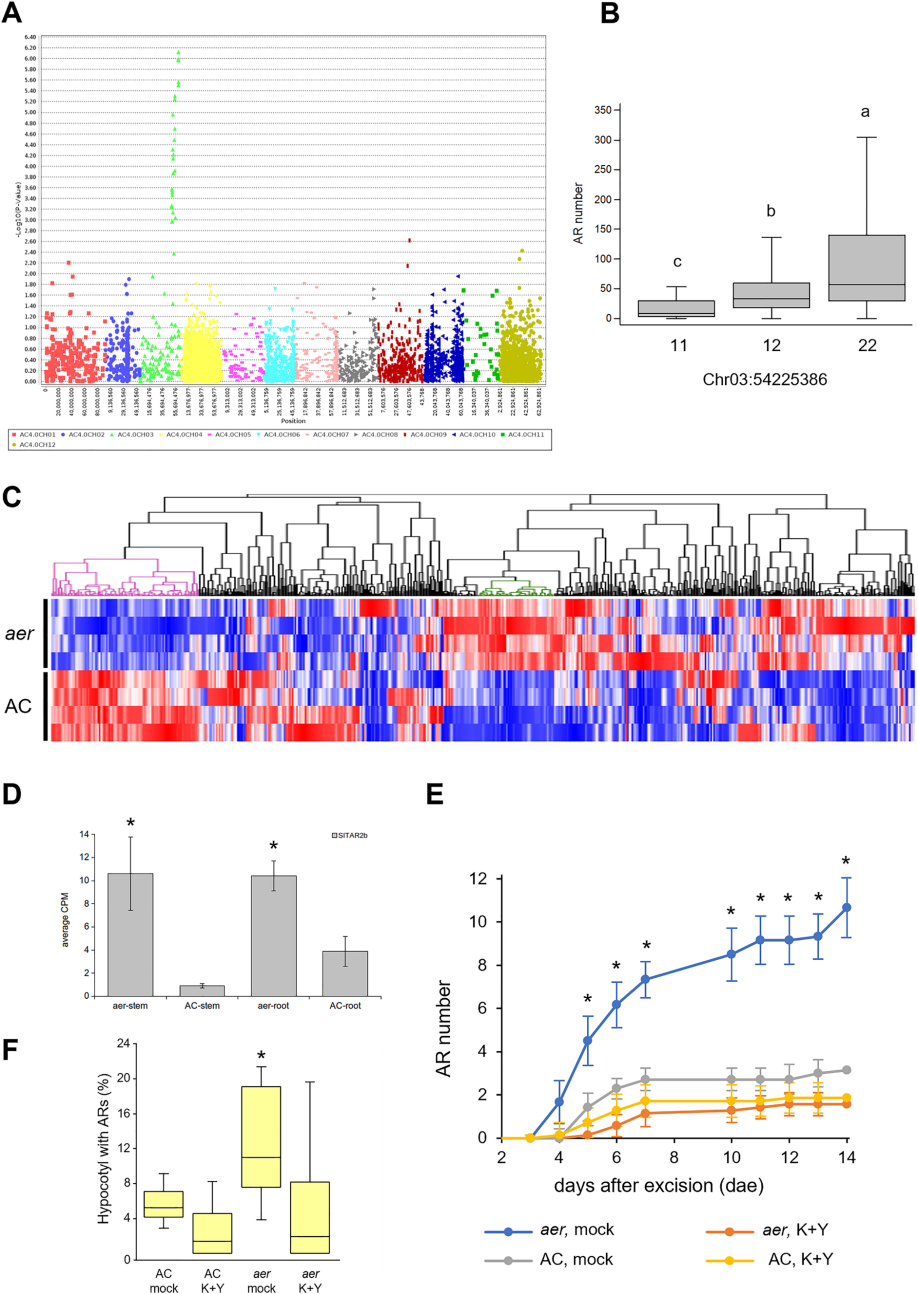
Identification of the quantitative trait locus (QTL) for AR formation in the *aer* mutant. **A** Manhattan plot of the QTL study for AR number from the F_2_ GBS population. **B** Box plot of AR number values used for genome-wide association study (GWAS), sorted by the genotype; 11 (AC homozygous), 12 (heterozygous) and 22 (*aer* homozygous), at SL4.0_chr03:54225386. **C** Hierarchical clustering of the average expression of the 593 genes expressed in AC and *aer* stem tissues located within the candidate interval containing the chr03-*aer* QTL defined by GWAS. Green and purple clusters represent highly up-regulated and down-regulated genes, respectively, in *aer* stem tissue compared to AC. **D** *SlTAR2b* expression in stem and roots. The expression was measured in three biological samples for each genotype. They were normalized to the expression level of *SlACTIN2* (*Solyc03g078400*) as a constitutive control. **E** Increase in the number of ARs in hypocotyl explants over the studied time. **F** Percentage of hypocotyl length with ARs at 7 DAE; K + Y: 50 µM L-kynurenine and 50 µM YDF. Letters/asterisks indicate significant differences (*p* value < 0.01) between samples

We generated an F_2_ population of 111 lines which was further studied by QTL mapping of the remaining loci controlling *aer* (Figure S2). A GBS approach was used to genotype this population and the data was associated with the AR phenotypes of the individual lines (Table S6A). Interestingly, the resulting Manhattan plot highlighted a strongly linked 10.4 Mbp genomic region on chromosome 3 (SL4.0_chr03: 49,227,396–59,648,347 bp) including 28 SNPs which were significantly associated with the AR^+^ phenotype (Fig. 7A, B and Table S6A, in italics). Detailed analysis of chromosome 3 haplotypes with high AR numbers in this population (lines #19, #40 and #63) allowed us to further reduce the *aer* region to ~ 6.2 Mbp (between 53,355,316 and 59,526,472 bp) (Table S6B).

The RNA-seq data of *aer* and AC stem and root tissues were analysed to identify deregulated genes within the isolated region. Hierarchical clustering analysis identified a subset of genes that were clearly upregulated in the *aer* stem compared with that of AC (Fig. 7C and Table S6C). Interestingly, among the four genes commonly upregulated in stem and root tissues of *aer* plants was *Solyc03g112460*, also named as *SlTAR2b* (Fig. 7D), a gene encoding the TRYPTOPHAN AMINOTRANSFERASE RELATED 2 (TAR2) enzyme involved in auxin biosynthesis (Ma et al. 2014). Indeed, *SlTAR2b* expression was specifically increased in the basal region of the hypocotyl after whole root excision of wild-type tomato shoot explants and was expressed at higher levels in the *entire* tomato mutant, where it contributed to the accumulation of new auxin maxima triggering wound-induced AR formation (Alaguero-Cordovilla et al. 2021). Previous results have determined that endogenous IAA accumulates in epicotyls and hypocotyls at higher levels in *aer* than in AC (Mignolli et al. 2017), which is consistent with our findings that the increased *SlTAR2b* expression in *aer* might enhance the AR numbers on the *aer* stem. We also found that genes assigned to the GO category “response to auxin” (GO:0009733) were significantly enriched in *aer* mutant tissues compared to that of AC (Table S6D).

To further confirm that the increased *SlTAR2b* levels might positively impact the number of ARs in *aer*, we treated *aer* shoot explants with L-Kynurenine, a competitive inhibitor of TAA1/TAR activity (He et al. 2011) and yucasin DF, a known inhibitor of YUCCA flavin monooxygenases involved in auxin biosynthesis downstream of TAR2 (Tsugafune et al. 2017). We found that treatment of young hypocotyl explants with these auxin inhibitors slightly reduced rooting capacity of AC (Fig. 7E), suggesting that local auxin biosynthesis had a limited effect on wound-induced AR formation in shoot explants with an endogenous auxin source, as described elsewhere (Alaguero-Cordovilla et al. 2021). Remarkably, these two inhibitors strongly and significantly reduced rooting capacity of *aer* shoot explants due to reduction in the size of the AR formative region (Fig. 7E, F), indicating that increased auxin biosynthesis in *aer* lines is required for the AR^+^ phenotype in *aer*.

### Genetic interactions between the three QTLs responsible for the* aer* phenotype

**Fig. 8:**
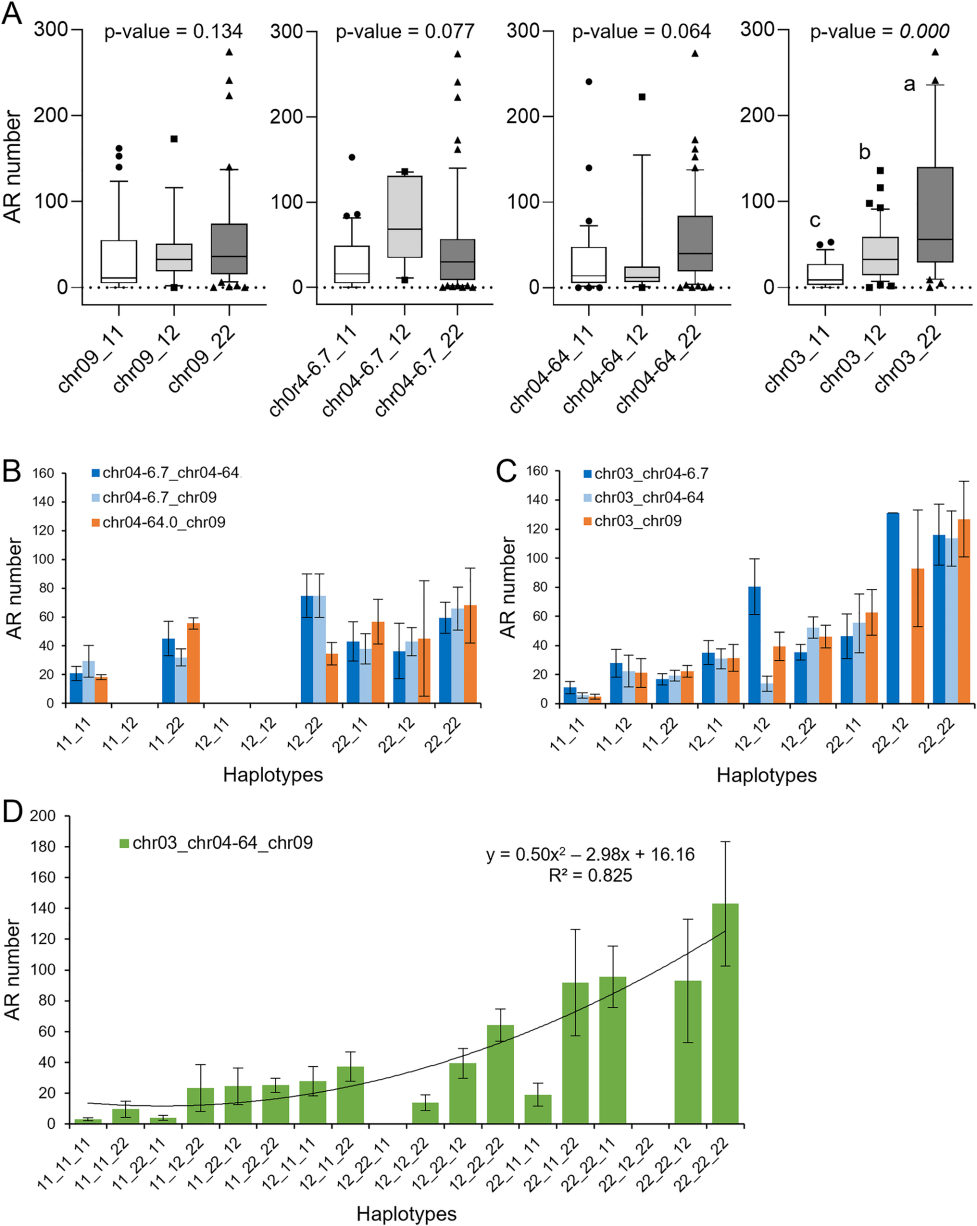
Interaction of QTLs involved in the formation of ARs in *aer* mutants. **A** Box plot of the number of ARs in the GBS population sorted by genotype, 11 (homozygous AC), 12 (heterozygous) and 22 (homozygous *aer*) at the indicated QTL markers. Letters indicate significant differences (*p* value < 0.01) between genotypes. **B**–**D** Number of ARs in lines with the same haplotype for the studied QTL. Bars represent the average ± standard error of at least three F_2_ lines in the GBS population with the indicated haplotype. Numbers in **D** indicate R-squared coefficient for a quadratic relationship between the two variables considered (number of ARs vs. number of *aer* alleles)

A large region of chromosome 4 was associated with the AR^+^ phenotype in the BSA mapping population spanning 57.3 Mbp (Figure S7). Two flanking KASP markers, chr04-6.7 and chr04-64.0, were used to genotype this region in the GBS population (Table S6E) and association studies were performed to evaluate the potential genetic interaction between the three genomic regions identified in this study to be associated with the AR^+^ phenotype of *aer* lines. We found that the QTL haplotypes on chromosomes 4 and 9 weakly contribute to the observed differences in AR numbers in this population when considered individually, and the major impact on AR numbers was controlled by the QTL on chromosome 3 (chr03-*aer*) (Fig. 8A). Furthermore, the combined effect of *aer* alleles at chr04-64.0 and chr09-*SBRL* considerably increased the number of ARs by fourfold (Fig. 8B). Interestingly, the chr04-6.7 QTL displayed heterotic behaviour either when studied alone or in combination with chr04-64.0 or chr09-*SBRL*, however, certain expected haplotypes were underrepresented in the studied GBS population (Fig. 8A, B). The heterotic effect of chr04-6.7 on AR number was further confirmed over *aer* alleles at chr03-*aer* (Fig. 8C). We found that *aer* alleles of chr04-64.0 and chr09-*SBRL* slightly but significantly (*p* value < 0.05) enhanced the effect of chr03-*aer* on AR number by 1.2- (chr04-64.0) and 1.3 fold (chr09-*SBRL*), respectively (Fig. 8B). Besides, a negative effect of chr04-64.0/chr09-*SBRL* AC alleles on chr03-*aer* homozygotes was observed, which was represented by the low AR numbers in lines #16, #48, #81 and #98 (Table S6E), indicating a complex interaction between these three loci. Lastly, the haplotype analysis provided strong evidence of the mutual interaction of *aer* alleles on chr04-64.0 and chr09-*SBRL* to quantitatively improve the effect of chr03-*aer* on AR numbers (Fig. 8D).

## Discussion

The *aer* tomato mutant was isolated in 1971 (LA3205) and depicted as a spontaneous mutant possessing unknown genetic background, which foreshadowed a complex genetic analysis. It was described as a *S. lycopersicum* line with ARs on the stem from soil level to a considerable height. AR production was later analysed under flooding stress, which induces ethylene insensitivity (Vidoz et al. 2016), and the PAT was reduced at the *aer* stem base, triggering AR initiation in the presence of additional auxin (Mignolli et al. 2017). Under typical greenhouse growth conditions, the *aer* line produces abundant ARs, not only in the basal region of the stem, but also in the upper parts, even on the fruit trusses, suggesting a significant accumulation of auxin throughout the stem (Fig. 1). Under the same growth conditions, Ailsa Craig and AC-*Tm-2*^*a*^ had no or very few ARs, making them ideal contrasting parental lines for genetic crosses and physiological studies.

During physiological analyses of *aer*, it was suggested that its AR phenotype may have a polygenic origin with epistatic interactions (Vidoz et al. 2016). We have used BSA to reveal these potential *aer* loci. We isolated two major regions on chromosomes 4 and 9, the latter with a highly significant BSA signal. Fine mapping of the *aer* phenotype to the distal end of chromosome 9 led to the isolation of the putative promoter region of the *Solyc09g066270* gene, which encodes the LATERAL ORGAN BOUNDARIES-DOMAIN 29 (LBD29) protein. *Solyc09g066270* has a predominant root- and stem-specific expression (Figures S9, S10) (Proost and Mutwil 2018), which can be significantly increased by auxin treatment (Figure S11) (Zouine et al. 2017). LBD29 is involved in LR founder cell acquisition (Lavenus et al. 2013) and is controlled by the auxin response factors ARF7 and ARF19, two transcriptional activators that directly regulate early auxin response genes, including *LBD29* (Okushima et al. 2007). In Arabidopsis, it is mainly ARF7 that binds to the *LBD29* promoter to increase its expression during LR development (Lavenus et al. 2015), while the transcription factors MYB94 and MYB96 can repress the expression of the *LBD29* promoter, thereby reducing callus formation (Dai et al. 2020). Furthermore, overexpression of *LBD29* in Arabidopsis increased the formation of LR primordia (Feng et al. 2012).

Tomato *LBD29* showed increased expression during wound-induced AR development in MT (Alaguero-Cordovilla et al. 2021) when root founder cell specification and root initiation occur at stages 2 and 3 of de novo root formation (Bustillo-Avendaño et al. 2018). We also tested the *aer* line for wound-induced AR production, and it produced significantly more ARs than the AC cultivar, indicating the involvement of LBD29 in the *aer* phenotype. A recent study investigated single cell transcriptomic profiling of transitional cell stages to reveal changes in gene expression during shoot-borne root formation (Omary et al. 2022). They examined factors with changing expression during the transition from phloem parenchyma to stem cells. Interestingly, *LBD29* was specifically associated with the cell transition state and showed a highly root-specific expression profile (Omary et al. 2022). CRISPR/Cas9 null alleles of *LBD29* were defective in shoot-borne and AR production in wound-induced hypocotyls, and the *LBD29* gene was later renamed to *SHOOTBORNE*-*ROOTLESS* (*SBRL*).

Similar to the AR-producing and wound-induced MT hypocotyls, the stem and root tissues of *aer* showed upregulated expression of *SBRL*. In the ~ 4.3 Kbp causative region, there was only one *aer*-related sequence polymorphism between *aer* and AC-*Tm-2*^*a*^, an “A” insertion that appears to be specific for *S. pimpinellifolium* (LYC2798, LA1584, LA1578) and *S. galapagense* (LA1044, LA0483) within the wild species sequenced, but also present in most sequenced *S. lycopersicum* cultivar genomes (47 out of 54) in the 150 Tomato Genome ReSequencing project database (Fernandez-Pozo et al. 2015), hence it seems to be a privileged allele in breeding programs. The mutation appears to be a potential binding site for the PLT2 transcription factor, which is essential for specification of the root quiescent centre (Aida et al. 2004) and plays a key role in the root regeneration process after root tip removal (Durgaprasad et al. 2019). It is also mentioned that PLT2 has an autoregulation mechanism during the establishment of root competence (Durgaprasad et al. 2019), which could be related to the *LBD29* promoter and its expression changes. Although the expression of *LBD29* is mainly controlled by ARFs and MYBs, the epigenetic regulation of *LBD29* through JUMONJI C DOMAIN-CONTAINING 30 (JMJ30) was also described in Arabidopsis (Lee et al. 2018), where JMJ30 act as a histone demethylase and it increases *LBD29* expression, which may be the case for *SBRL*. However, *SlJMJ30* localized to chromosome 1 (*Solyc01g006680*) which was not detected in the BSA or QTL analyses of *aer*.

Although the impact of *SBRL* expression on the *aer* phenotype is ensured, the mutant parental line produced significantly more ARs than subsequent F_2_ recombinants containing the mutant *SBRL* alleles. This effectively suggests the presence of other loci that further impact AR numbers. BSA of *aer* line revealed a large region on chromosome 4 that could also influence the extreme AR phenotype. With two flanking markers, F_2_ lines carrying *aer* alleles on chromosome 4 were selected and produced significantly increased number of ARs when carrying the mutant *SBRL* allele. A multiple genome comparison using the Tersect pipeline (Kurowski and Mohareb 2020) in the region between 6.7 and 63.3 Mbp on chromosome 4 (SL4.0) showed high homology between *S. pimpinellifolium* and *aer*, which was not the case for most of the *aer* genome (Figure S8). Although there is no evidence that *S. pimpinellifolium* produces more ARs than AC or other cultivar lines, it definitely has a higher LR initiation capacity than certain cultivars (Alaguero-Cordovilla et al. 2018), which could contribute to the increased RSA of *aer* (Fig. 2**.**). The contributing region of *aer* on chromosome 4 was considerably large, so it was unlikely to identify the causative genes in this region. However, the AR phenotype of the combined *aer* QTLs confirmed that contributing loci on chromosome 4 could be located closer to 64.0 than to 6.7 Mbp.

Surprisingly, the coexistence of *aer* alleles at loci on chromosomes 4 and 9 was still not sufficient to restore the extreme AR phenotype of *aer*, suggesting that further loci were involved in the phenotype. During the BSA of the *aer* × AC-*Tm-2*^*a*^ cross, we clearly identified two major regions contributing to AR development on chromosomes 4 and 9, but there was no further significant BSA peak to localize more loci for *aer*. On the other hand, the large BSA peak on chromosome 9 overlaps with the *Tm-2*^*a*^ introgression of *S. peruvianum* in AC-*Tm-2*^*a*^ and the long causative region of *aer* on chromosome 4 appears to be related to *S. pimpinellifolium*. These two regions with a distant genetic origin possess abundant polymorphisms between the parental lines, which can lead to recombination “cold spots” (Chetelat et al. 2000; Fuentes et al. 2022) and result in very few recombination events through these regions (Figure S5). The pool of 45 AR^+^ lines did not show the extreme AR phenotype of *aer*, since the additional causative loci segregated randomly in the F_2_ lines. If the loci on chromosomes 4 and 9 contain dominant or epistatic genes and the additional locus is recessive, then it will be difficult to detect a contrasting BSA peak between the two pools of ~ 50 lines. Therefore, the phenotype selection of ~ 50 lines in each BSA pool could have been biased and more associated with these larger non-recombinant chromosomal regions than with other, more recombinant causative regions, which loci could be underrepresented and go unnoticed behind dominant BSA signals on chromosomes 4 and 9.

In order to reveal the further loci influencing the *aer* phenotype, we analysed an additional F_2_ population which revealed a strong peak on chromosome 3 including approximately 2000 genes. In this F_2_ population, the QTL analysis did not detect the causative loci on chromosome 4 and only a moderate QTL peak was found on chromosome 9 (Fig. 7A). Nonetheless, the 111 F_2_ lines were preselected for homozygous alleles at loci on chromosomes 4 and 9. Consequently, the number of lines with heterozygous alleles at these loci was lower than in a typical segregating population, most of these alleles were fixed (Table S6E). This biased population could cause distortion of potential QTL peaks along the chromosomes 4 and 9, while the rest of the recombinant genome segregated randomly, enabling us to detect the additional locus on chromosome 3.

*SlTAR2b* (*Solyc03g112460*) was one of the few genes that were upregulated in both stem and root tissues of *aer*. In Arabidopsis, *TAR2* is expressed in the root pericycle and vasculature, and its overexpression causes an increase in the number of LRs, while the knockout mutant shows reduced LR development under low nitrogen conditions (Ma et al. 2014). *SlTAR2b* is an essential gene for the tomato auxin biosynthesis pathway and shows increased expression in the basal region of hypocotyl explants after wounding (Alaguero-Cordovilla et al. 2021). Indeed, the reduced activity of SlTAR2b by chemical inhibition prevented wound-induced AR initiation in MT shoot explants (Alaguero-Cordovilla et al. 2021), and we found that it also efficiently reduced AR formation in the *aer* lines. This strongly suggests the implication of SlTAR2b in the initiation of the strong AR phenotype in *aer*.

On the other hand, another auxin-related gene, *SlARF9* (*Solyc03g113410*), was also located in the QTL region of chromosome 3. Although transcriptional studies and transgenic experiments with promoter-GUS fusions of *SlARF9* showed its expression in primary root and LR meristems, the main function of ARF9 is related to fruit development (de Jong et al. 2015). The *SlARF9* gene has comparable expression levels in *aer* and AC, further excluding its involvement in the *aer* phenotype.

Surprisingly, the *SlPIN1* (*Solyc03g118740*) gene was also located near the causative region of chromosome 3. PIN1 is one of the major regulators of PAT in plants (Okadalat et al. 1991) and the *SlPIN* genes showed increased expression during AR initiation in *aer* using RT-PCR experiments in young seedlings (Mignolli et al. 2017). In our RNA-seq experiments, *SlPIN1* did not show expression differences between the AC and *aer*, but the plants we used here were more developed, 4 weeks old (the AR primordia were already present). Similar to *SlPIN1*, the up-regulated *SlPIN3* (*Solyc04g007690*) and *SlPIN4* (*Solyc05g008060*) genes previously reported in the hypocotyl (Mignolli et al. 2017), also showed no expression differences between the AC and *aer* in the 4 week-old stem and root tissues.

AUX/LAX proteins also play a central role in auxin distribution (Kramer 2004), and expression analysis in tomato suggested SlLAX2, SlLAX4 and SlLAX5 might be involved in PAT in stem tissues (Pattison and Catalá 2012). In *aer* hypocotyls, the expression of *SlLAX4* (*Solyc10g076790*) and *SlLAX5* (*Solyc10g055260*) are decreased compared to AC and suggest reduced PAT towards the roots (Mignolli et al. 2017). Our results, however, showed no significant differences in expression in 4-week-old lines. In contrast, SlLAX3 (*Solyc11g013310*) had significantly increased expression in *aer* (Figure S12). While the transcriptional factor ARF7 controls the *LBD29* expression, LBD29 binds to the *LAX3* promoter to control its transcription which then leads to LR emergence (Porco et al. 2016). Although there was no difference between AC and *aer* in *SlARF7* (*Solyc07g042260*) expression in the stem tissue, there was a slight but significantly increased expression of *SlARF7* in the roots. This may explain why the *aer* lines also had a more vigorous root system than AC in the growth experiments (Fig. 2). The root phenotype of the GBS population (Figure S2) indicated variable RSA segregation among the F_2_ lines (Figure S13). Due to the complex genetic background of *aer*, we were unable to investigate the phenotype link between the strong AR traits and the underground basal or LR systems of this population. Nevertheless, the first observations on the phenotype segregation of LRs and ARs suggested that the genetic control of the two root types is not entirely the same. One might need to create an extended segregating population to test AR and LR phenotypes simultaneously, which is a rather challenging analysis and beyond the scope of this study.

We demonstrated that the extreme AR phenotype of *aer* is driven by high local auxin accumulation in the stem, which is generated by increased activity of SlTAR2b as a key regulator of the process. In turn, SlTAR2b will contribute to the high expression of *LBD29*/*SBRL*, which will lead to increased activity of SlLAX3, resulting in excessive induction of ARs. The combined use of genomic tools (BSA, GBS and transcriptomics) uncovered a functional relationship between three QTLs on different chromosomes for the extreme AR formation phenotype in *aer,* but the causative genes on chromosomes 3 and 4 need to be identified by subsequent fine mapping or reverse genetic approaches.

During the grafting process, the scion’s ability of wound-healing and tissue regeneration is more critical than high AR production on the rootstock’s lower stem. This study identified genes and alleles of *aer* that can directly support breeding strategies for clonal propagation of tomato or other species. To improve nutrient uptake, it may also be beneficial to consider different combinations of independent *aer* causative loci. The identification of the remaining causative alleles responsible for the *aer* phenotype would greatly assist in achieving more finely-tuned and advantageous AR phenotypes compared to the combined and severe AR production of the original mutant line.

### Supplementary Information

Below is the link to the electronic supplementary material.

Supplementary file 1 (DOCX 3553 kb)

Supplementary file 2 (XLSX 38 kb)

Supplementary file 3 (XLSX 53 kb)
