# Supplementary material for: Genes involved in auxin biosynthesis, transport and signalling underlie the extreme adventitious root phenotype of the tomato aer mutant
Source: Theor Appl Genet. 2024 Mar 8;137(4):76. doi: 10.1007/s00122-024-04570-8 (PMC10923741; doi:10.1007/s00122-024-04570-8)
Supplement: Supplementary file 1 — (DOCX 3553 kb) [file 122_2024_4570_MOESM1_ESM.docx]

# **Supplementary Figures**


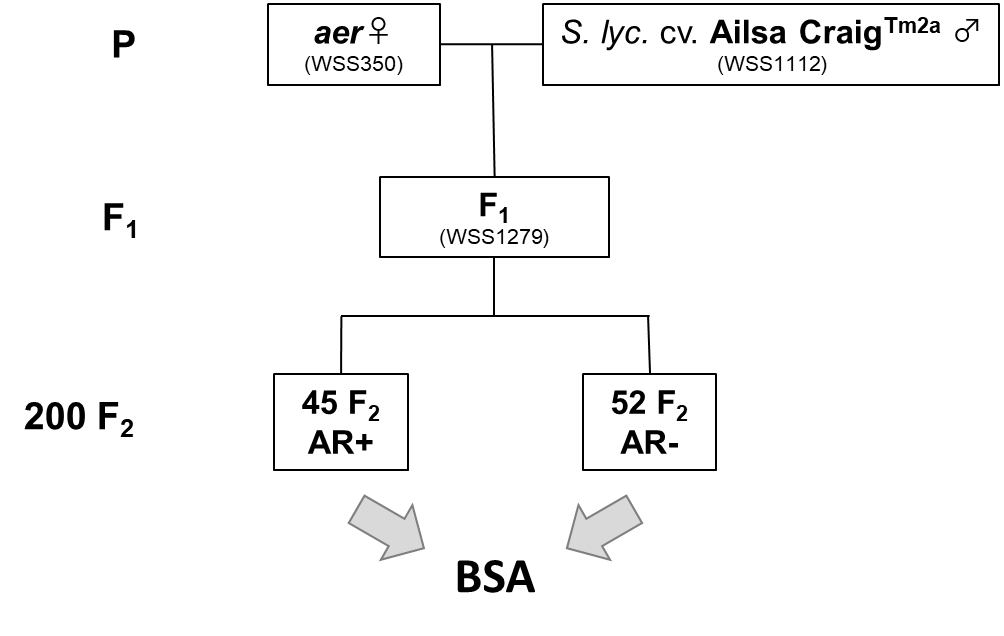


## **Figure S1**. **Pedigree of the *aer* mapping population used for BSA and fine mapping**. WSS numbers are the Cranfield seed accessions used for the study. The number of F_2_ AR^+^ and AR^-^ lines were used for BSA are indicated.


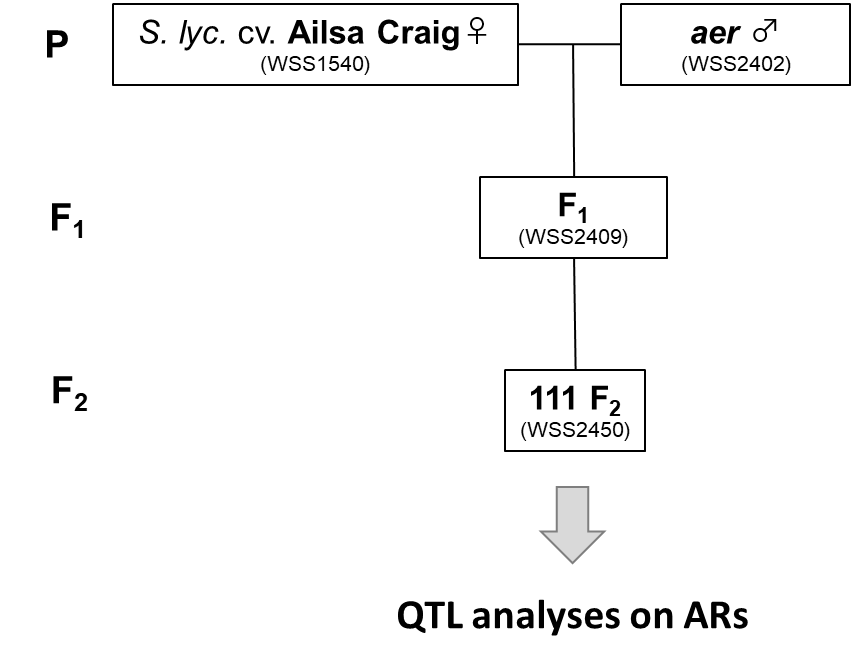


## **Figure S2. Pedigree of the *aer* mapping population used for QTL analyses for the additional causative loci**. WSS numbers are the Cranfield seed accessions used for the study.


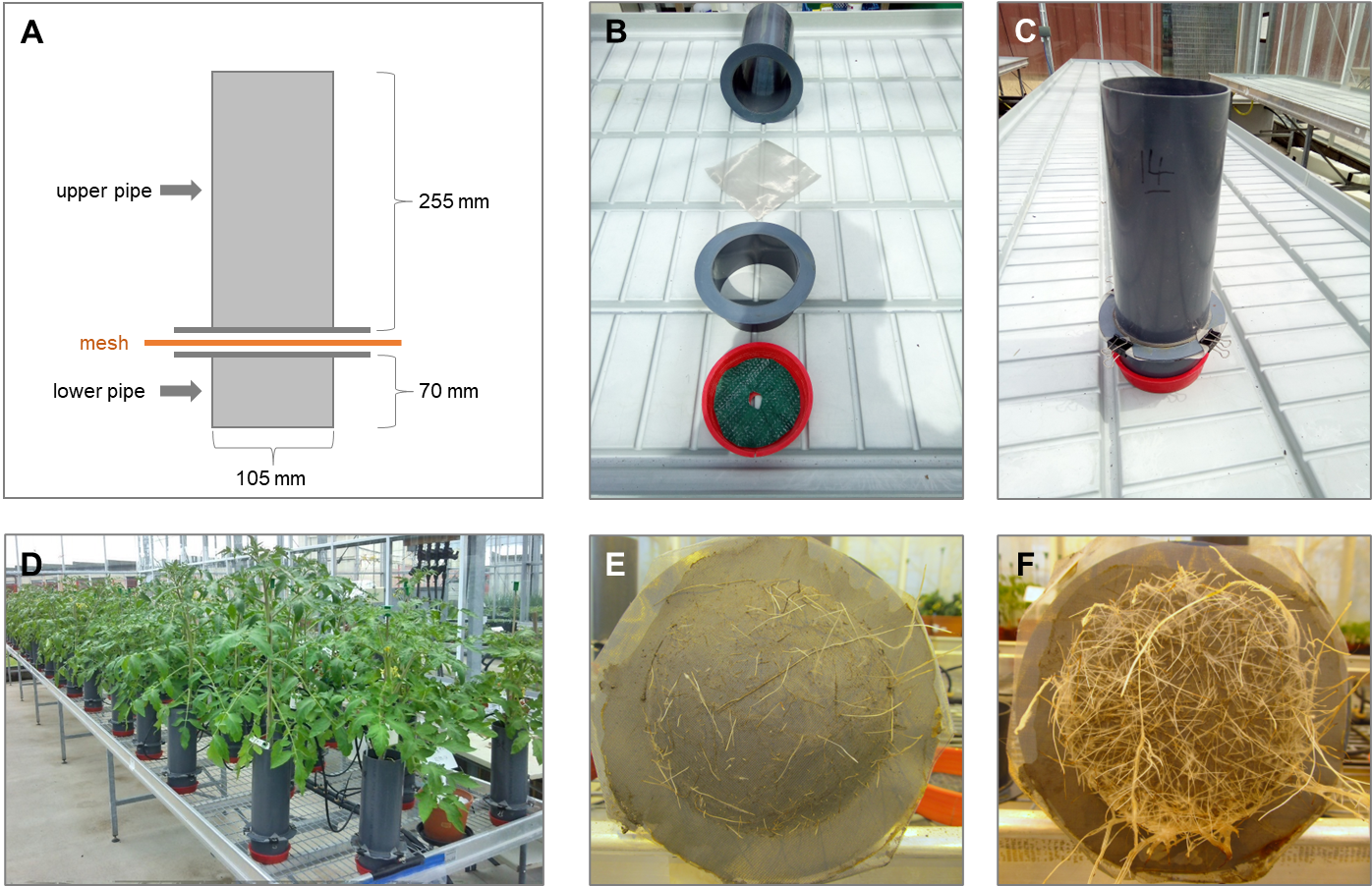


## **Figure S3. Root penetration assay**. (**A**) the structure and dimension of assay pipes including a dividing mesh are described. (**B**) the parts and the (**C**) assembled system were used in the glasshouse in (**D**) randomised pattern. (**E**-**F**) the different genotypes produced different penetrating root growth.


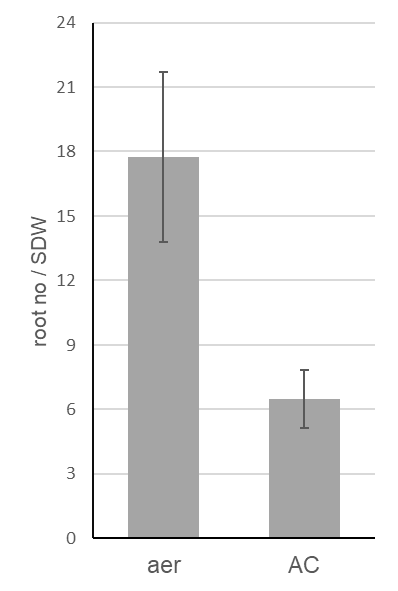


## **Figure S4**. **Penetrating root numbers.** The shoot dry weight (SDW) related to the number of penetrating roots of the two genotypes shows a significant difference. Six replicates of *aer* and AC were used for counting and analysed by one-way ANOVA (LSD: 5.108, p< 0.001), standard deviation values are marked.


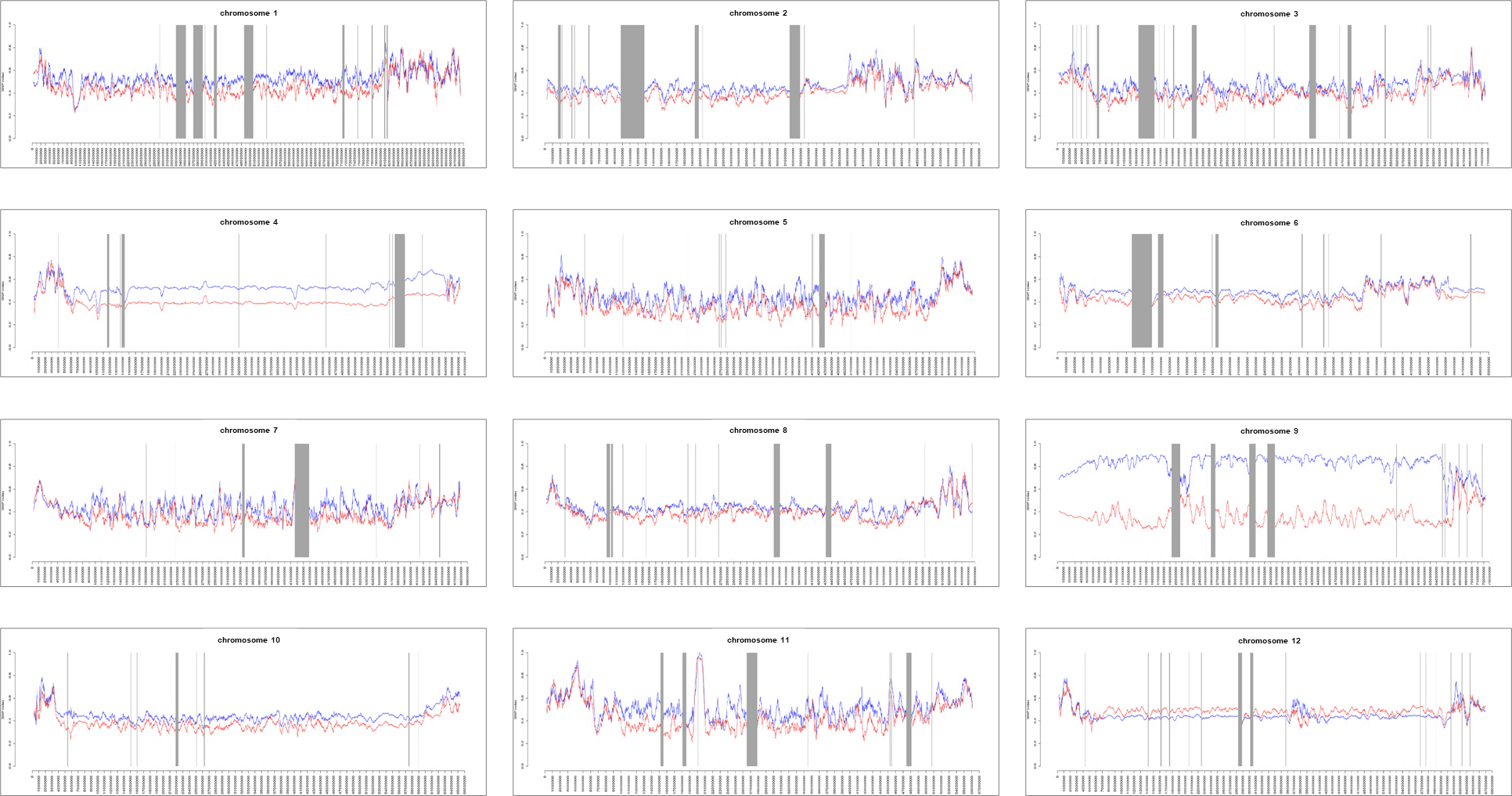


## **Figure S5. SNP zygosity index of AR^+^ and AR^-^ bulked pools.** The SNP distribution compared to Heinz 1706 genome are marked in with blue for AR^+^ and red for AR^-^ pools along the 12 chromosomes. “1” value corresponds to homozygous SNPs, “0.5” means heterozygous SNPs at given base pairs. Grey columns represent sequence gaps in the SL2.50 reference genome.


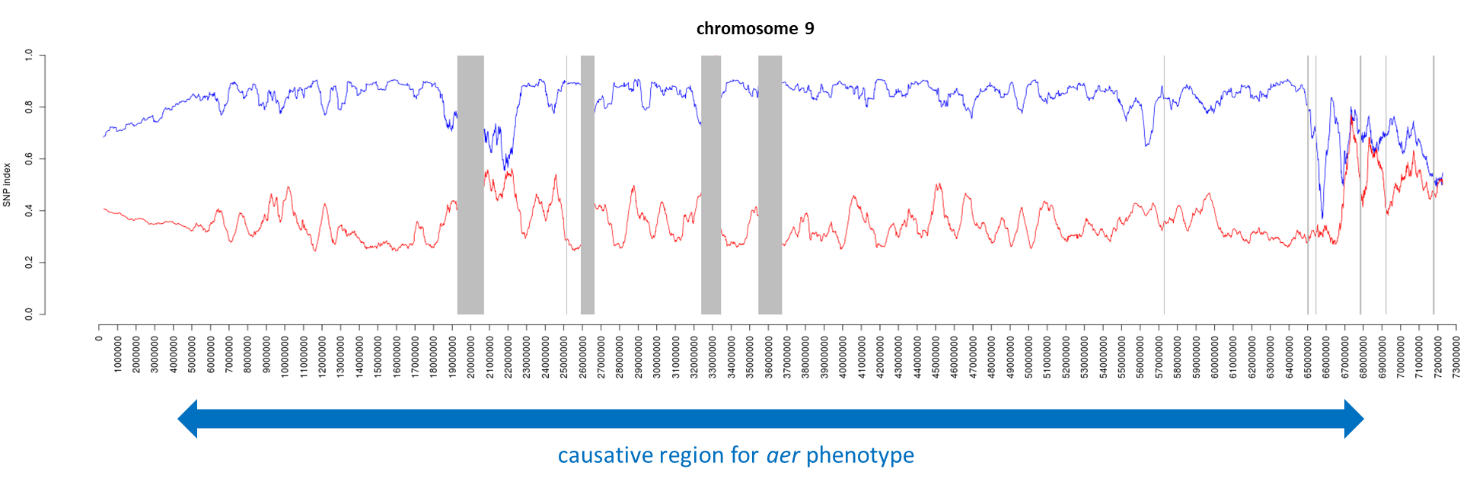


## **Figure S6.** **SNP zygosity index of AR^+^ and AR^-^ bulked pools on chromosome 9**. The SNP distribution compared to Heinz 1706 genome are marked in with blue for AR^+^ and red for AR^-^ pools. “1” value corresponds to homozygous SNPs, “0.5” means heterozygous SNPs at given base pairs. Grey columns represent sequence gaps in the SL2.50 reference genome. The potential causative region for the *aer* phenotype is delimited between 4.0 and 68.0 Mbp.


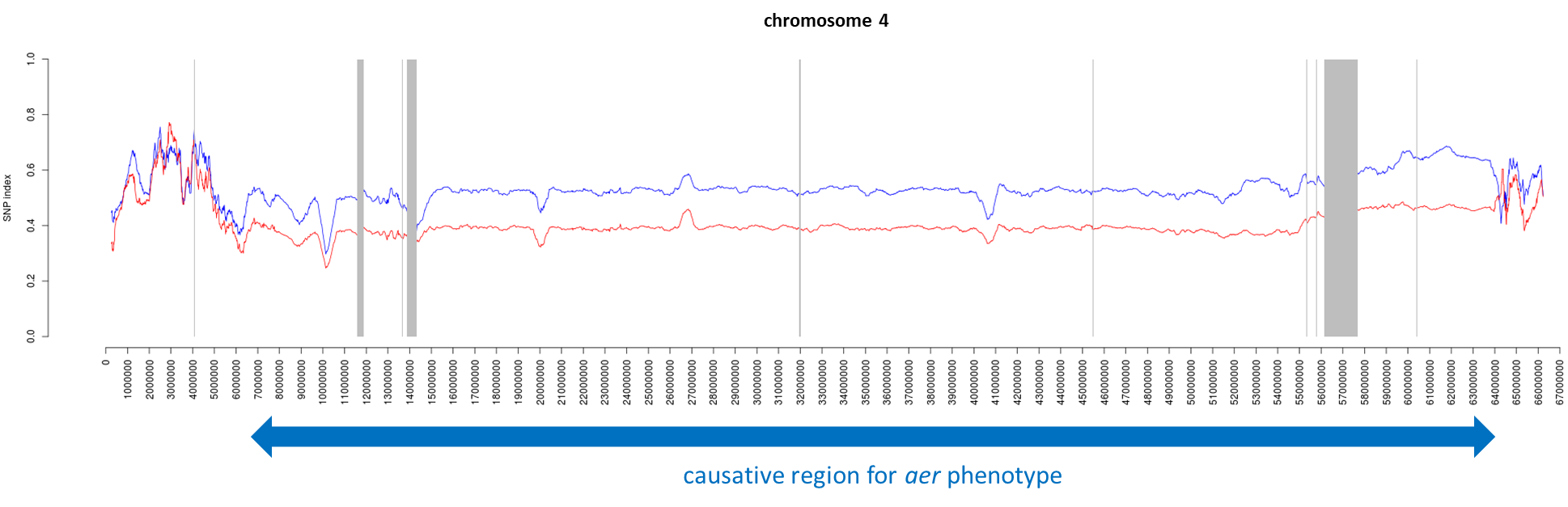


## **Figure S7.** **SNP zygosity index of AR^+^ and AR^-^ bulked pools on chromosome 4**. The SNP distribution compared to Heinz 1706 genome are marked in with blue for AR^+^ and red for AR^-^ pools. “1” value corresponds to homozygous SNPs, “0.5” means heterozygous SNPs at given base pairs. Grey columns represent sequence gaps in the SL2.50 reference genome. The potential causative region for the *aer* phenotype is delimited between 6.7 and 64.0 Mbp.

**
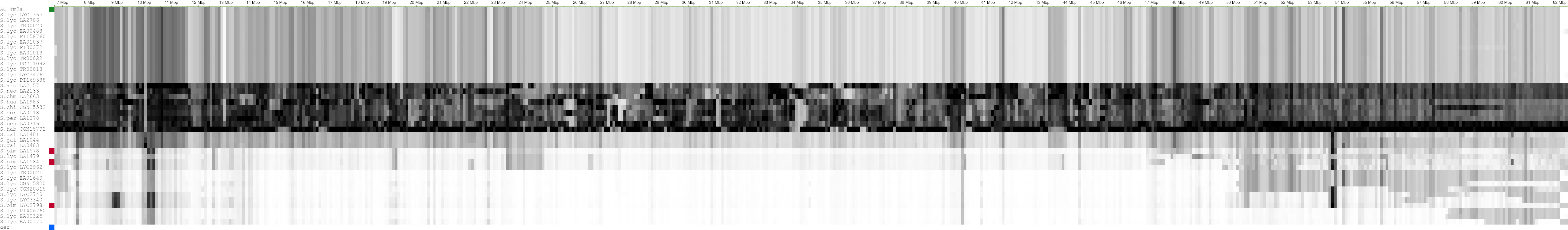
**

## **Figure S8.** **Potential *S. pimpinellifolium* introgression region on chromosome 4 between 6.7 and 62.3 Mbp (SL4.0) in *aer***. AC-*Tm-2^a^* (marked with green) is closely homologous to the presented 13 *S. lycopersicum* cultivars (top), while the *aer* (blue) genome is more homologous to other cultivars but also closely related to certain *S. pimpinellifolium* lines (red), especially to LYC2798 on chromosome 4. Less related wild species are also included.

**
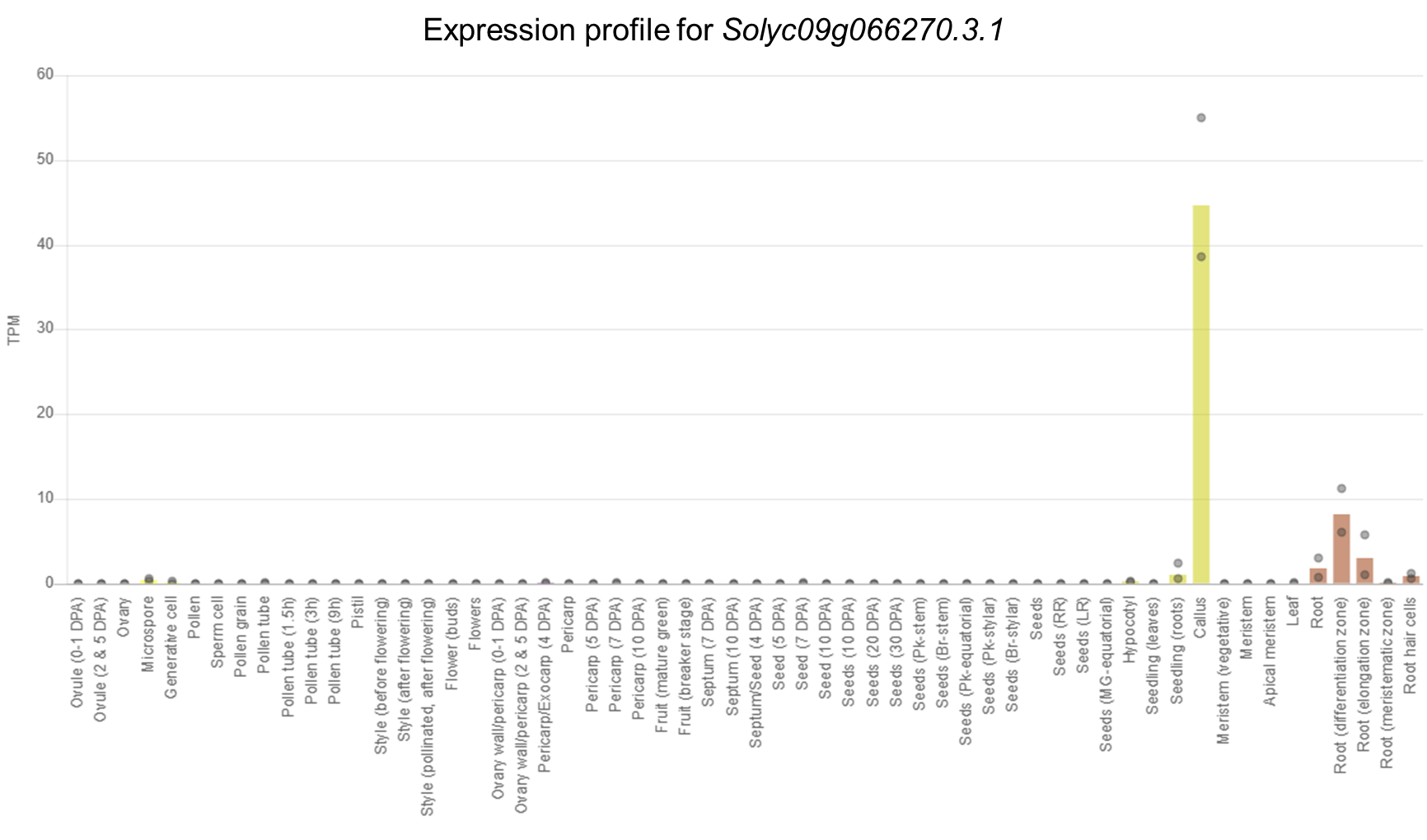
**

## **Figure S9**. Expression profile of *Solyc09g066270* in the CoNekT RNA-seq database using “all conditions”. Expression values are given in transcripts per kilobase million (TPM), normalised for read count and gene length. Bars represent mean value; circles represent minimum and maximum values.

**
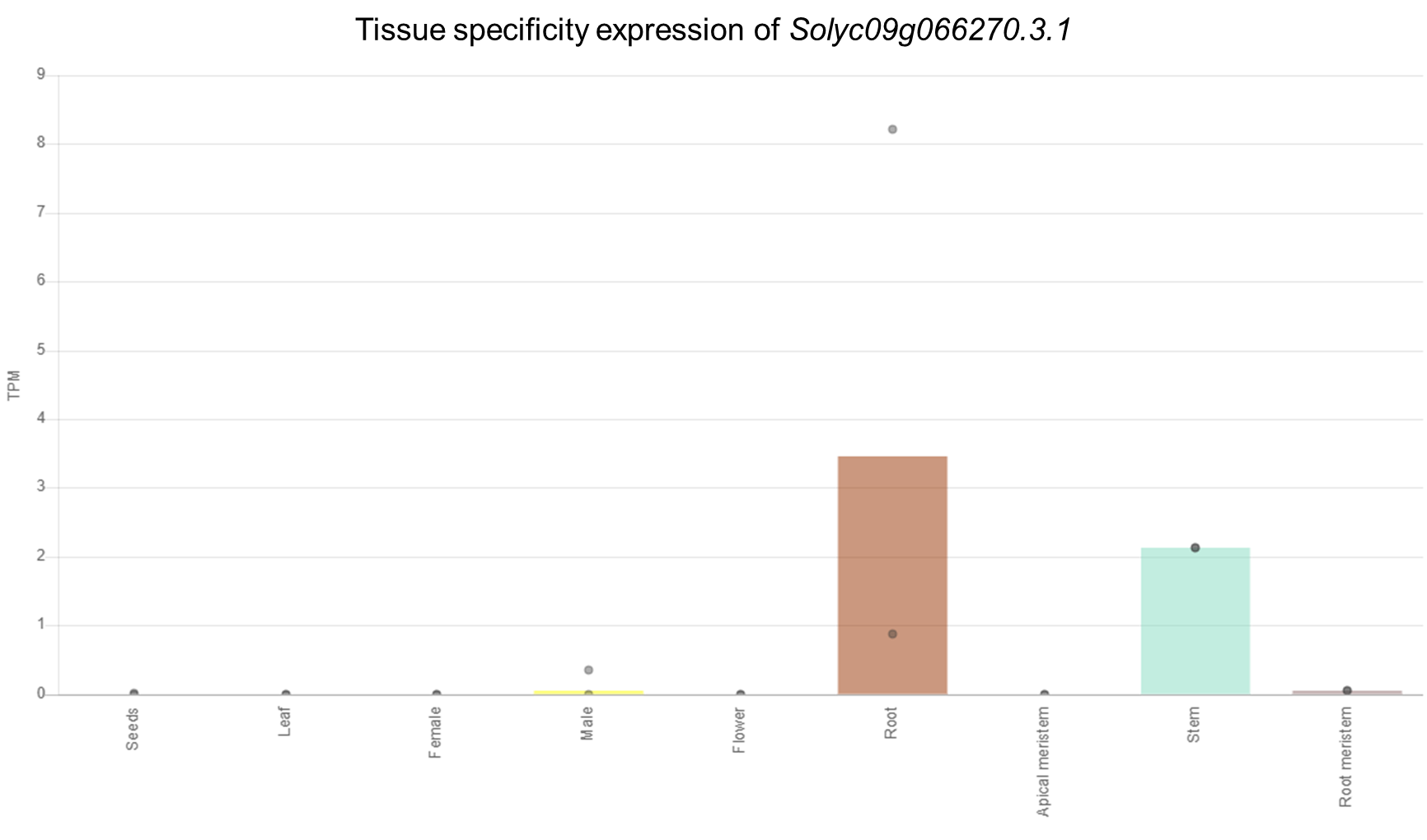
**

## **Figure S10**. Tissue specific expression profile of *Solyc09g066270* in the CoNekT RNA-seq database. Expression values are given in transcripts per kilobase million (TPM), normalised for read count and gene length. Bars represent mean value; circles represent minimum and maximum values.

**
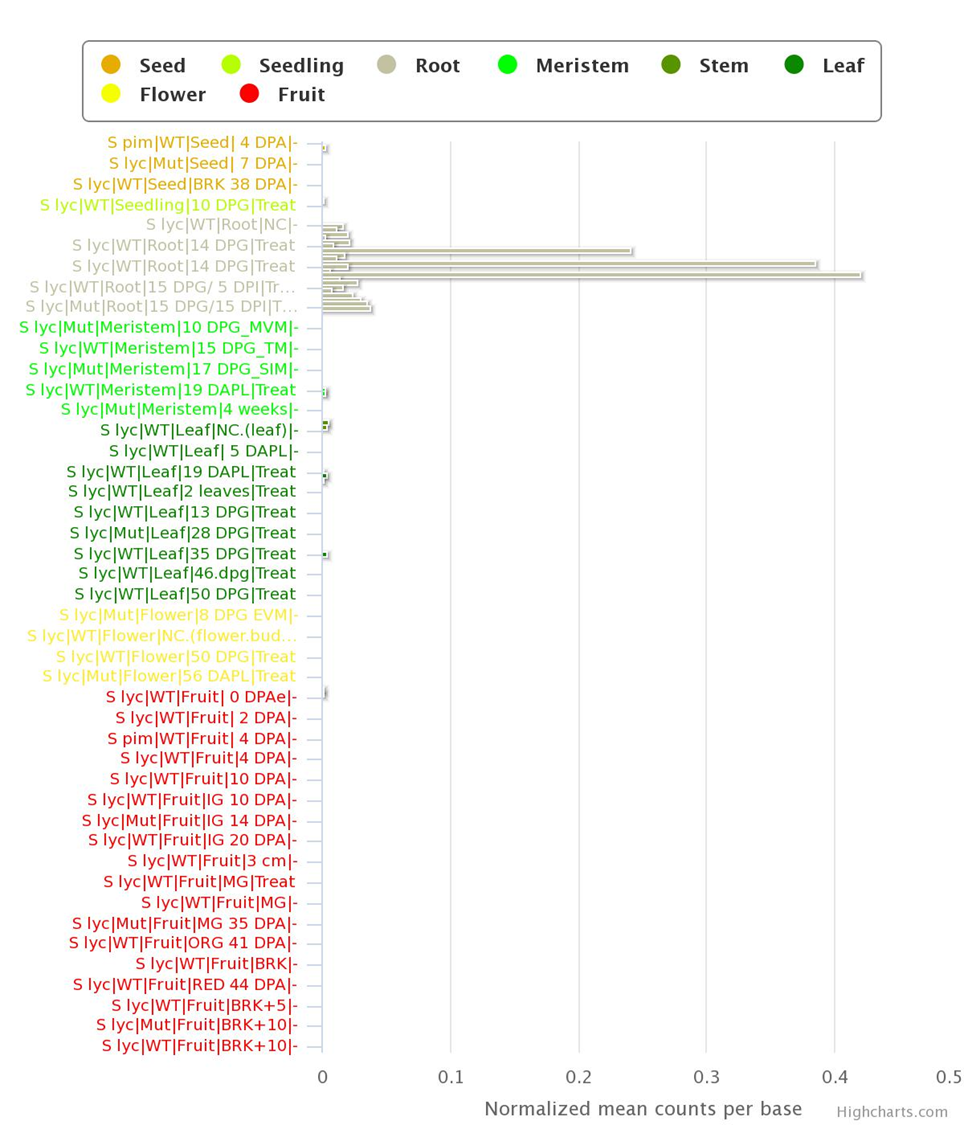
**

## **Figure S11. Tissue specific expression profile of *Solyc09g066270* in the TomExpress RNA-seq database**. Bars are colour coded; grey stands for significant root specific expressions, including 3 samples with extreme values, when root samples were treated with auxin for 24h.


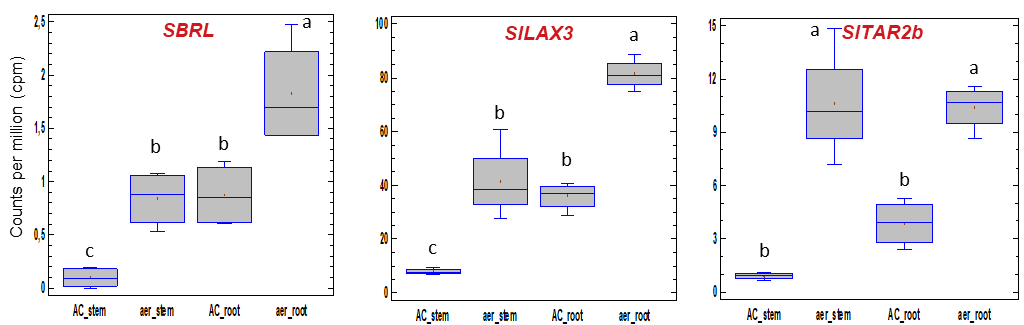


## **Figure S12. Expression of candidate genes related to auxin that contribute to the AR^+^ phenotype in *aer* lines**. Letters indicate significant differences (p-value < 0.01) between samples.

**
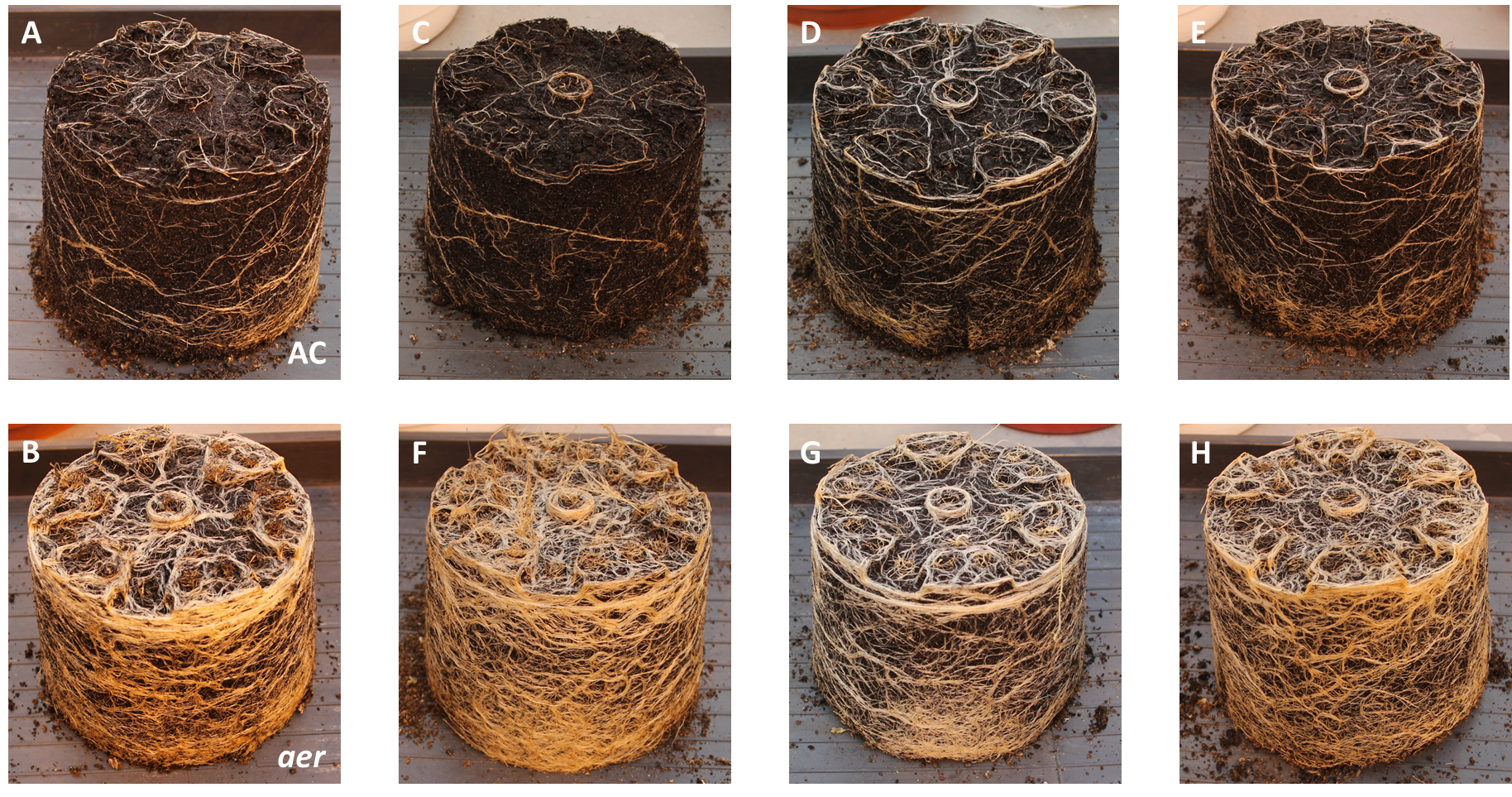
**

## **Figure S13. Root vigour segregation of *aer*.** The RSA of the parental AC (**A**) and *aer* (**B**) lines are shown by the soil blocks in the pots at the end of the growth experiment. The segregating F_2_ population (Figure S2) lines presented a large RSA variation, a few extremes like the parental lines are shown on **C-E** and **F-H**.

# **Supplementary Tables**

## **Table S1 Sequences submitted to LGC Ltd for design of KBD assays**.

| **marker name (SL2.50)** | **SL4.0 position (bp)** | **sequence for KASP design** |
| --- | --- | --- |
| chr9_457,550 | 459,004 (chr9) | TCATAAAAAGAATTCTACTAGATGCGTGTCATCAGTACACTTGAACCTAACGCTCTGATACCAACTTTGTCATGATCCAAGTCTATACCATAATCAAGACAGGGTACTTGGAACCACAAGTAATCTCAAATTAACCATTGTCATACATATAAAATCATAACGTAAAG[G/A]AACCAAGCAAAATCTGAAATTCATAAGCGATTAATAAAATCTAAAAAATTCATGAAACTATATTTCTAACTATTTTAAAAT |
| chr9_4,551,475 | 4,585,437 (chr9) | CACTGGTGGATTTGGTGAAGTATTTAAAGGTATTTTAGATGATGGTACTATAGTTGCAGTTAAAAGAGCAAAACCCGGTAACGCTAAGGGCACGTTACAAGTCCTTAATGAAGTTCGAATCTTGTGCCAAGTGAACCATCGTGGTCTAGTAAAATTACTCGGCTGTTGTGTCGAGCTCGAATTACCTCTTTTAATTTACGAGTACGTACCTAACGGTACGCTTTTCGAGCACCTCCACGTGTTCCGTCTCCGCGGGC[A/G]GGCCCCACTTAGTTGGTTGAGGCGTCTCGTGATCGCTCAACAAACCGCGGATGGGCTCGCGTACCTACACTCATCGGCCGTTCCGCCGATTTATCATCGCGATGTCAAGTCGAGTAATATTTTACTAGACGATAAGCTCGATGCTA |
| chr9_12,780,335 | 12,816,435 (chr9) | CCTAACCCCTTCCCTTCGGAATAACTTGAAACCCTTACCTAGAATCGAATTGGTTTCGTAGATTAATAATTGGTTTCCTAGTTATCCTAAAAATTAGGTGGCGACTCTTTTTTAAACTCGTTTAATTTAACTTTTGTTAAAATTGAAACAATTAATTATTTTTGAGTTGTCGCGACGTTTCGCCCTATTTTGACTGAATAGGGATGTAAACAAAAGTCATAGTTTATGAGTCTAGGAATAAATCTCCACCT[A/C]CTCCACCTTTCGAGAGTAATAGATGGTMTCCATTAATGATCCAATTGAGGCTTCCTAGAAACAAACCACTCCACACTCTTCTRCGCACGTGAGAGA |
| chr9_24,824,514 | 23,805,259 (chr9) | CAAAAGAGCATGTTTTATATAAATTTCCTTTTAGCATGATTTTATTGCATATCACCTACTTAGTACATTCAATGTGCTAATTCCATA[T/C]GTTGTTTTATCTTTAAAGGTRTAGGTGGAATTAGAGAGGAGTCATTTGAAGTGAAGCTCGAAAACTAGCTGTCACGACCCAAACCGGGTTGCGACTGGCACCCACACTTACCCTCCTATGTGAGCGAACCAACCAATCTAAATCTTAACATTTCAATGTAATAACAACAGAAAATAATTCGGAAGACTTAAACTCATTAATAAAACCAATTCAATAACTATCAATATTCAACATCTATTA |
| chr9_50,136,746 | 46,233,608 (chr9) | GAAACTTATCAGAGCATATGCAAAGTTGTAGAGCTAGCAGGACATTGTGCAGCTAGGGAACCATCTCAGAGACCAGATATGGGCCACGTGGTAAATGTCTTAGCTCCTCTGGTAGAGCAATGGACACCCACGGCC[A/G]CTGCTGGAGATGATAGTTTCAATATTGACTTCACTATGAGCCTTCCTCAAGCCCTGCAAAAATGGAAAGCTAATGACAATTCCATGCTCTCTGAAGACACGTCGTATGGTGACTATAGC |
| chr9_58,607,422 | 54,682,397 (chr9) | GCTTGCCCATAAATTGATGAAGGATCCATGGGCAMAGACTTGTGAGTGCCACCCAAGTTCACTTCCCCTTTAATATCCTACATAGTTGAAATACGTATCG[T/C]CATTAGCTTGGTAAAAGAAGACAAAATACCGAGTATTGAACAAAGAAGAGATATCCTGTCTAGTATCTAGAACTAAGAGCTCATGTAGTTACAGTTGGCATCTGAGGCCTTCCTTGTATTTGCTGCAACATGTTCCCTGAATTTCCTTGTACCAACTGGCTGAATCAACAGAGAAGAAATAGAGATACAAATGATTCAAAGCATGCAACAAATAGTTACGTGTTGTGTAAAATAATGCAATAACATA |
| chr9_59,613,138 | 55,693,834 (chr9) | TGCTTCCACTTCTTGTATGCACTGTTATTTTCCAGTCCGTCAGCAGGAAGACTTGTKTCATTCCCATTAACAACATCCCACAAATCCTCTCTCACAAGGTATGATCCCATACAT[A/G]TCTTCCATACCTTGTAATTGGACTGATTCAACAACTCCATCCCCAGTCCATTAACACGACATCTAAAATCCATATCGACAAACCAGTTGAATCTACCACTAAC |
| chr9_60,614,863 | 56,694,927 (chr9) | CTTTAAGGTCCTCCTCAAGGACCMTTAGGGTGGTCCTTGGAGGTTYGTACCTTGGCGTCTAACCCATAAACACCTCAACCATGACTCGAGACAACATCAAACACCTA[C/G]AAAACTCAAGACAAACTCACACACTTACTAATTAGGCTCTAGTTTCACTAATTCAATTWTTAGGGTCGTTACAAGGGTGAGTCTTATTAACCTTATTTACTTTCGTATGTTTTAATAATGATAA |
| chr9_62,963,512 | 59,062,287 (chr9) | GGTGAATGTGAGACATGAAATAATGGATCTTGAGCAGTGGTAGAATCAATACTGTTGGTTTGAAAAGGGAACTCCTTCTCACAAAAGRTTACATCCCTGCTAACAAAAATAGTCTTGGATTCAAT[A/G]TCAAACAATTTGTACCCCTTTTGACTGACAACATAACCAAGTAACACAGCCTTCCTTGCCTTTGCTGCAAATTTATCATCTTTTCTCAAATTTGTAGCAAAA |
| chr9_63,992,176 | 60,090,876 (chr9) | TCATAGTACTTAAAATTTTAAAATAACGGCAATTAAAAAAAAACTTTTACCACACTAATTCAACAAAATAACTGCACCACACTAAAAATTATCTCAATTAAAATTATCCCATTTTCTTCTATTTTCTTCTGGTTTTCCTATTCAATAATATAACAAAATTTAGATACTCCTAGTA[C/A]GTAAATACAATTAGAGGGGGTATGAGTACTAAAAYCGATCAACAAATTGAATTG |
| chr9_64,696,470 | 60,793,971 (chr9) | GAACCTAATGACAATACATAAAAATCCATCATCAACACAAAATATTGATAAATTCTACACATCATCATGAT[G/C]TTTTGTGCAAGATTCTTCCTTTGCGGCTTGCAGGATTCTAAGAAGCAGAACCTGACATTTACAGTCTATTGTTGACTGA |
| chr9_64,698,737 | 60,796,234 (chr9) | GTGGATCATAAAATATTGAATACTAAATTAACTTAATTGATAGTGGTTCCCTTATTGGGCTACATTTTTATTTATTTTTTGTAATATTAAATTCTCTTTACCCCCTTACATTCTCAAATTAACATCCTTAAAGGACAAAAGAGGTAGCAAAAACCAAACCCATACATCTATGCTTGCTGCTAATTCATCACT[A/C]AGCTTATCAATGTCTTTCAATTGATAACAACATTCATATTTGGGAGACGTTTGATCATGAAAATTTGTCACTTTTC |
| chr9_64,716,346 | 60,813,518 (chr9) | TTTATCGTCCTCACACACTGCTGAAATAAATAAAATGAGCAACTTGTTTGKTGAACAGAAAACCTTTGAAACATCAGTATCCTGACCTCGGTCTGACTAG[A/T]AAAATGTATGTCTATTCTGACCTTCCAGCTCTACAAGTTGATCTTTGCYTGYAGTAGCTGCATAAGAGACCATAATACATCCATGCGATGCAGGGTTCTATC |
| chr9_64,929,454 | 61,028,434 (chr9) | ACTATCATTATTTTATAAGTTATTTTACTGCTTTTTAATTTATCAAATTAGAATAACTAGTAGTATTACTAGGTAATACTAT[T/C]AATAGGTAGAAACTTTTGTCCTTATAAACCAATTGACCATTTTRCTTTRTGTTTTTTGAATGTTAAAGCAACTCAAAGGGTATTATCGAGGATAT |
| chr9_72,389,099 | 68,460,785 (chr9) | CTACACGAAAAAGAACCACACACTACCATATGATTTATAGTTTTACGTTTATCGATTTTTTAACCATATATATCGCAAAATTATAAGGAATAAATGAAAGTTAAAT[C/A]AGATAAATGGAATGAGTTTACACATAGATCTCAAAATAATAAGCAACTTTTAAAAAAGAATCGCCTCCAAATGATACTCGAACAAAAAGTTACAGCCATTTGAAGTTTCAACAACTGTAATGTTAAAGGGGTAGTTGTGAAAATTTGAACTTCAAACTGAACGGACGTCCACAAAAAAAATTGGCATTTTTGACGTCGGAATCCGGATCACCCAAAAAATGGTTTCTATAGC |
| chr4_6,783,452 | 6,815,697 (chr4) | GCTTCTTTGCATTCTTTTACGTTTGCATGCAATTAAAAAAATATTCTGATGCTAAAGAAAGTACTGCATATTTAATAGTTGAAACAAAGTCGCAACTTCTGTTGAGAGTATACTCAATATCATAAGGTTAGTTTTGAGTTGCTGAGAAAACTAGTAACGTTTTTGGTGAGTTATTGATCTTGACATGATTTATAATGATGAACAAACTTGTGTTCTATTCTTATTTCGGAGTCAGTTTCTACTTTCA[A/G]TTGACTAATTCATGTTTGTTCTGGTTTCTTAAACTGTATTTTTAGGCATAATTGTCAACGCTCTAGCTGAAAGAATGGTTGACATGAAGGAAAACATTGGCAATAACTTTTTCGCTTCTTTTCAGAATGGGAATACGGTATGTATGCTTCTGACTG |
| chr4_64,028,212 | 62,005,329 (chr4) | CTATGTTCCAATTTAATCTTTACTTTTCTCTAATGATAATTCTGTCTCACCTTCTTTGTATGTCAAACCAAAATTTAAGATGAACACCAGAGGTAACAATTATTCAATCCAATACTTGGAAACGATTGATTTATCA[G/C]AAGATTACTCAGTCAATAGTATATATATTACTTCTTGATTATGATTTTTTTTTCAACAAAGAAAATATATAAGTTTATGAAATAATAATTATTAGATGATCAGACAAGAAATTTTAGTTGAAAAAACAAATAAACAAAGCCAACAAAA |

SNPs/InDels were detected by whole genome sequencing (SL2.50) of parental lines (AC-*Tm-2^a^* and *aer*) and were used to design KASP markers. Polymorphism positions in SL4.0 are also included.

## **Table S2. Primer sequences of larger InDel markers on chromosome 9**

| InDel position in bp (SL2.50) | forward primer | reverse primer | SL4.0 position (bp) |
| --- | --- | --- | --- |
| 64,204,608 | AAGTCGATTCATAATGCCTC | AATTATGCAGGGGTTCACTG | 60,303,182 |
| 64,431,713 | GAGTTAAATAGTACATGATC | CTCTCTACGTCAGATAGGCG | 60,529,924 |
| 64,587,053 | CTACGTAACTATCGACACATG | CAGGCACTAACGCTCGTACTC | 60,685,339 |
| 64,639,488 | AATGACTATCTAATGACGGGC | ATATACTTCTGAAAGACCCC | 60,737,629 |
| 64,646,938 | ATCTATAGTTTGCTTTCTCC | ACAAATAGTACGTATTTCTC | 60,745,059 |
| 64,680,847 | CAAGAACTCAAGGGGTCGGC | AATACGATCTTTCCTTGCAC | 60,778,973 |
| 64,693,928 | AAGAGCTATAATGGGTAGGC | GGTGAAAACTCAAGGCTAC | 60,791,430 |
| 64,703,104 | GTATTTTATCAAACTATC | CTTGAATATATTCAGAATCTAC | 60,800,603 |
| 64,710,070 | GACAGAAATACATACATA | CTCAAACAACGTCCATGTC | 60,807,247 |
| 64,743,835 | CTTCTATATTCTATCCTATCC | TCTAAGACATCAGTTTTACC | 60,840,466 |
| 64,751,084 | CTCACTTCTCACTAGGAAGC | CCACTTTAGAAGTGACTTC | 60,847,720 |
| 64,754,113 | CAGCCCATAGATATAACATG | GATGAAGTTTTGGCATGAC | 60,850,756 |
| 64,770,771 | ACAATTACAATTTACATGTG | GAATGATTGAATTCTAGTGAC | 60,867,349 |
| 64,781,470 | CAACACTCCATCCAATACGAG | ATAAACATTTAACCATCTCC | 60,878,119 |
| 64,789,251 | AGCTAGACACTAATCATGCA | CTAACATGAGCCAAACTATT | 60,885,419 |
| 64,878,712 | GATCCTCATGAAAGGGTGAGG | GTGTCATCCATTCGAGGTGGC | 60,975,844 |

InDels were detected by whole genome sequencing of parental lines (AC-*Tm-2^a^* and *aer*) using SL2.50 tomato reference genome. InDel positions in SL4.0 are also included.

## **Table S3. AR numbers and genotypes of key recombinants (F_3_ generation) for mapping of *aer* chromosome 9 loci**

## **Table S5. Genotypes and AR numbers of F_2_ lines were used for mapping the chromosome 4 loci of *aer***. 11 (homozygous AC) and 22 (homozygous *aer*) representing the selected alleles on chromosome 4 (combined selection with chr04-6.7 and chr04-64.0 markers) and chromosome 9 (chr09-*SBR*).

| **F2 plant #** | **chr4** | **chr9** | **combined** | **AR number** |
| --- | --- | --- | --- | --- |
| 2 | 22 | 22 | 22_22 | 44 |
| 23 | 22 | 22 | 22_22 | 13 |
| 36 | 22 | 22 | 22_22 | 16 |
| 92 | 22 | 22 | 22_22 | 44 |
| 107 | 22 | 22 | 22_22 | 20 |
| 110 | 22 | 22 | 22_22 | 1 |
| 3 | 22 | 22 | 22_22 | 304 |
| 54 | 22 | 22 | 22_22 | 274 |
| 9 | 22 | 11 | 22_11 | 9 |
| 53 | 22 | 11 | 22_11 | 5 |
| 61 | 22 | 11 | 22_11 | 0 |
| 21 | 22 | 11 | 22_11 | 9 |
| 50 | 22 | 11 | 22_11 | 42 |
| 78 | 22 | 11 | 22_11 | 116 |
| 80 | 22 | 11 | 22_11 | 162 |
| 86 | 22 | 11 | 22_11 | 57 |
| 105 | 22 | 11 | 22_11 | 140 |
| 11 | 11 | 22 | 11_22 | 0 |
| 109 | 11 | 22 | 11_22 | 11 |
| 75 | 11 | 22 | 11_22 | 33 |
| 16 | 11 | 11 | 11_11 | 5 |
| 48 | 11 | 11 | 11_11 | 0 |
| 81 | 11 | 11 | 11_11 | 15 |
| 98 | 11 | 11 | 11_11 | 11 |
| 34 | 11 | 11 | 11_11 | 5 |
| 99 | 11 | 11 | 11_11 | 5 |
| aer_1 | 22 | 22 | 22_22 | 445 |
| aer_2 | 22 | 22 | 22_22 | 523 |
| aer_3 | 22 | 22 | 22_22 | 562 |
| aer_4 | 22 | 22 | 22_22 | 583 |
| AC_1 | 11 | 11 | 11_11 | 3 |
| AC_2 | 11 | 11 | 11_11 | 1 |
| AC_3 | 11 | 11 | 11_11 | 0 |
| AC_4 | 11 | 11 | 11_11 | 2 |
